# Omipalisib reduces hyperphosphorylated tau protein by modulating mTOR-autophagy pathway

**DOI:** 10.1371/journal.pone.0352120

**Published:** 2026-06-23

**Authors:** Haeun Hwang, Namkwon Kim, Subyn Jeon, Yoojin Lee, Jeongmin Son, Seung Ho Jeon, Yeongae Lee, Min Sung Gee, Kyung-Soo Inn, Jong Kil Lee

**Affiliations:** 1 Department of Fundamental Pharmaceutical Science, College of Pharmacy, Kyung Hee University, Seoul, Republic of Korea; 2 Department of Pharmacy, College of Pharmacy, Kyung Hee University, Seoul, Republic of Korea; 3 Department of Neurology, Yale School of Medicine, New Haven, Connecticut, United States of America; 4 Department of Neurobiology and Behavior, University of California, Irvine, California, United States of America; 5 College of Pharmacy and Institute of Integrated Pharmaceutical Sciences, Kyung Hee University, Seoul, Republic of Korea; University of Florida, UNITED STATES OF AMERICA

## Abstract

Tauopathies are neurodegenerative diseases characterized by the presence of hyperphosphorylated tau (p-tau) and neurofibrillary tangles. Autophagy is a critical self-degradation mechanism that preserves cellular homeostasis and function, including the clearance of misfolded proteins. Autophagy is impaired in tauopathies, resulting in excessive accumulation of p-tau. Omipalisib, a dual phosphatidylinositol 3-kinase/mammalian target of rapamycin (PI3K/mTOR) inhibitor, was explored in a phase I clinical trial involving solid tumors and lymphoma. In this study, we aimed to investigate the effects of omipalisib on tauopathy both *in vitro* and *in vivo*. Omipalisib increased the levels of protein LC3B and decreased that of p62 in human tau (P301L)-expressing SH-SY5Y stable (SH-Tau) cells by inhibiting mTOR activation in a time-dependent manner. In our study, we hypothesized that omipalisib, a PI3K/mTOR inhibitor, could remove accumulated tau and inhibit memory decline by activating autophagy. Additionally, omipalisib reduced tau phosphorylation in SH-Tau cells without inducing cytotoxicity. Upon administration of 6-month-old PS19 mice with omipalisib (1 mg/kg) for 2 months, the levels of both RIPA-soluble and RIPA-insoluble p-tau were decreased, and spatial memory dysfunction was alleviated in omipalisib-treated PS19 mice. Overall, these results show that omipalisib decreases the expression of p-tau by modulation mTOR-autophagy pathway, resulting in the amelioration of spatial memory deficits. This study highlighted the potential of omipalisib as a candidate treatment for tauopathies.

## 1. Introduction

Tau is a soluble protein that binds to microtubules and is important for axon growth and microtubule stabilization and assembly [1]. Under pathological conditions, structural changes such as phosphorylation and acetylation of tau disrupt the binding of tau to microtubules, making it unstable, leading to its dissociation from the microtubules, further driving the progression of the disease [2]. The aggregation and accumulation of these abnormal tau proteins in nerve cells are characteristic of tauopathies such as Alzheimer’s disease, progressive supranuclear palsy, and corticobasal degeneration [3]. Given its role, tau is identified as a major target in these diseases. Consequently, many studies have focused on preventing tau deposition or removing the accumulated tau using various strategies, including microtubule stabilization, inhibition of tau aggregation, regulation of post-translational modifications, regulation of exon 10 splicing, tau immunization, and increase in tau clearance [3].

In humans, degradation pathways such as the ubiquitin-proteasome system and autophagy-lysosome pathway (ALP), facilitate the removal of abnormal proteins; however, these pathways are dysregulated by the excessive accumulation of pathological proteins, including modified tau [4]. ALP dysfunction is a pathological feature of tauopathy [5,6]. Several studies have demonstrated that autophagy activators decrease phosphorylated and aggregated protein levels. For example, Schaeffer *et al*. reported that autophagy stimulation by trehalose reduces the amount of insoluble tau protein and improves neuronal survival in the brain of a P301S tau mouse model [7]. Ozcelik *et al*. showed that rapamycin reduced insoluble tau protein levels by promoting autophagy via inhibiting the mammalian target of rapamycin (mTOR) activity in the P301S tau mouse model [8]. These results highlight the potential use of autophagy activators as treatments for tauopathies.

Omipalisib, a dual inhibitor of phosphoinositide 3-kinase (PI3K) and mTOR, has been subjected to early-phase clinical trials to determine its safety, tolerability, and pharmacokinetics. These studies have revealed its substantial inhibition of the PI3K/mTOR pathway in patients [9]. In preclinical studies, omipalisib exerted anticancer effects by inducing DNA damage and apoptotic signaling via PI3K/mTOR pathway inhibition [10,11]. Moreover, in head and neck squamous cell carcinoma, omipalisib increased the mTOR-mediated autophagic response, which in turn reduced cell proliferation [12]. Based on the involvement of ALP in tau clearance, we hypothesized that induction of autophagy by omipalisib could relieve tauopathy. To this end, we treated mutant tau-overexpressing cells with omipalisib and used the PS19 mouse model of tauopathy.

## 2. Materials and methods

### 2.1. Materials

**Fig 1:**
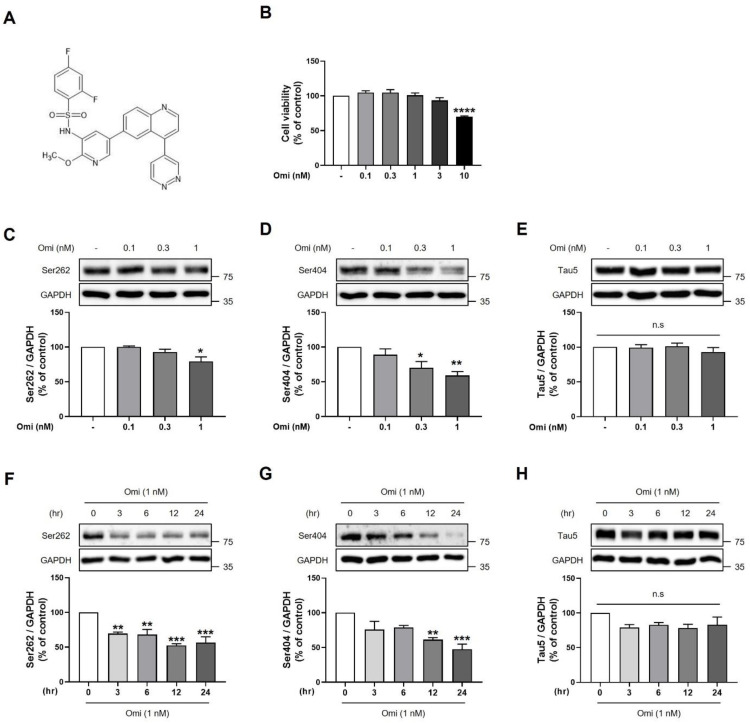
Effects of omipalisib on the overexpression of phosphorylated tau in SH-Tau cells. **(A)** Chemical structure of omipalisib. **(B)** Effect of omipalisib on cell viability in SH-Tau cells. Cells were treated with different concentrations (0–10 nM) of omipalisib for 24 hr. Cell viability was measured with a WST-1 assay. The levels of Ser262 **(C)**, Ser404 **(D)**, and Tau5 **(E)** in whole cell lysates were measured by western blot assay. **(F-H)** Time-dependent reduction of p-tau expression by omipalisib. SH-tau cells were treated with omipalisib (1 nM) for different durations (0–24 hr). The levels of Ser262 **(F)**, Ser404 **(G)**, and Tau5 **(H)** in whole cell lysates were measured using western blot assay. GAPDH was used as an internal control. Statistical analysis was performed using one-way analysis of variance and Dunnett’s post hoc test. Data represent the mean ± SEM of three repeated experiments. *p < 0.05, **p < 0.01, ***p < 0.001, and ****p < 0.0001 compared to the control group. n.s, non-significant.

Omipalisib (C_25_H_17_F_2_N_5_O_3_S, HY-10297, > 99% purity, Fig 1A) was purchased from MedChemExpress (Monmouth Junction, NJ, USA). Paraformaldehyde (PFA), phosphate-buffered saline (PBS, pH 7.4), Triton X-100, dimethyl sulfoxide (DMSO), and Tween 80 were purchased from Sigma-Aldrich (St. Louis, MO, USA). Roswell Park Memorial Institute media (RPMI) was purchased from Welgene (Gyeongsan, Republic of Korea). Fetal bovine serum (FBS), GlutaMAX, and penicillin-streptomycin (P/S) were purchased from Gibco (Waltham, MA, USA).

### 2.2. Cell culture

P301L (0N4R) mutant tau-expressing SH-SY5Y cells (SH-Tau; P30705) were purchased from Innoprot (Ibaizabal Bidea, Spain). Cells were cultured in RPMI containing 10% FBS and 1% P/S, GlutaMAX, and 125 μg/mL G418 at 37 °C in a humidified atmosphere of 5% CO₂. After stabilization for 24 hr, cells were treated with 0.1, 0.3, or 1 nM omipalisib.

### 2.3. Cell viability

A water-soluble tetrazolium (WST-1) assay was performed to assess omipalisib cytotoxicity. SH-Tau cells were seeded at a density of 8 × 10^4^ cells/well in 96-well plates. Subsequently, the cells were treated with 0.1, 0.3, 1, 3, and 10 nM omipalisib for 24 hr. WST-1 solution was added to each well and incubated for 2 hr. The absorbance of the cells was measured at 430 nm using a FLUOstar Omega microplate reader (BMG Labtech, Ortenberg, Germany).

### 2.4. Animals

We conducted a pilot tolerability study evaluating omipalisib at 1 and 3 mg/kg and monitored body weight, and clinical condition. Animals were monitored at least once daily, and predefined humane endpoints included severe lethargy, inability to access food or water, marked weight loss (>20% of baseline body weight), persistent hunched posture, or severe respiratory distress. Animals meeting these criteria were immediately removed from the study and euthanized. In the 3 mg/kg group, 3 of 7 mice reached humane endpoint criteria and were euthanized, whereas no animals in the 1 mg/kg group reached humane endpoints or died (S1 Fig). Based on these observations, 1 mg/kg was selected for the main experiment. All animal procedures were performed by trained personnel experienced in laboratory animal care and handling.

Wild-type (WT) and tau P301S (1N4R) mutant (PS19) male mice (aged 6-month-old) were purchased from Jackson Laboratory (Bar Harbor, ME, USA). Experimental mice (*n* = 40) were divided into four groups based on the following treatment: WT+vehicle (10% DMSO and 5% Tween-80 in saline-treated WT mice), WT+omipalisib (omipalisib 1 mg/kg-treated WT mice), PS19 + vehicle (vehicle-treated PS19 mice), and PS19 + omipalisib (omipalisib 1 mg/kg-treated PS19 mice). Vehicle or omipalisib (1 mg/kg) was intraperitoneally injected into the mice once a day for 2 months (S2 Fig). Body weight of the mice was measured weekly throughout the treatment period to monitor any potential changes (S3 Fig). During the 2-month treatment period, no animals reached humane endpoint criteria. The animals were housed in plastic containers (2–4 mice per container), with controlled temperature (23 ± 1 °C), humidity (60 ± 10%), a 12 hr light/dark cycle, and ad libitum access to food and water. Mice were assigned to experimental groups using a block randomization method. To minimize bias, all behavioral testing and quantitative analyses were conducted under blind conditions. The mice were anesthetized by intraperitoneal injection of avertin. For western blot analysis, euthanasia was performed by cervical dislocation under deep anesthesia. For immunofluorescence, mice were euthanized by transcardial perfusion with PBS followed by 4% PFA fixation. All procedures were carried out under anesthesia to minimize pain and distress. All animal studies were performed in accordance with the “Guide for the Care and Use of Laboratory Animals, 8th edition” (National Institutes of Health, 2011) and the ARRIVE guidelines. The study was approved by Kyung Hee University on August 9, 2023 (approval number: KHUASP(SE)-23-523).

### 2.5. Morris water maze test

To evaluate spatial learning, the Morris water maze (MWM) test was performed, as previously described [13]. The MWM test was conducted in a white tank (1.0-m diameter, 30-cm height) filled with water to a depth of 20 cm (22–24 °C). A white, opaque powder was added to conceal the platform in water. The mice were acclimatized one day before training, followed by testing three times per day, in one of four quadrants. The time taken by the mouse to swim to the hidden platform was recorded, up to a maximum of 60 s. If the mouse did not mount onto the platform within 60 s, it was guided to the platform and allowed to remain there for 10 s. After 7 days of training, the platform was removed, and a probe test was performed. Mice were allowed to swim freely for 60 s. Data was collected for the time the mouse required to traverse the cleared platform area and the time spent in each quadrant. All tests were recorded using a video monitor and a camera connected to a computer.

### 2.6. Preparation of RIPA-soluble and insoluble tau proteins

The brains of the mice were harvested, weighed, and suspended in a 12 × volume of radioimmunoprecipitation assay (RIPA) lysis buffer (Thermo Fisher, Waltham, MA, USA) and protease/phosphatase inhibitor cocktail. The brains were homogenized and incubated on ice for 30 min. Subsequently, the homogenates were centrifuged at 13,000 rpm at 4 °C for 20 min. The supernatants were collected as RIPA-soluble fractions and stored at −80 °C for further analysis. After centrifugation for 20 min, the pellets were washed once with RIPA buffer and incubated with 2% sodium dodecyl sulfate (SDS) solution at room temperature (RT) for 1 hr followed by centrifugation at 15,000 rpm for 1 min. The collected supernatant was categorized as RIPA-insoluble fractions.

### 2.7. Western blot analysis

Western blotting was performed as previously described [14]. An equal amount of protein lysates (10 or 20 µg) was quantified by Bradford assay. Equal amounts of protein were separated by SDS-polyacrylamide gel electrophoresis and transferred to polyvinylidene difluoride membranes. The membranes were washed five times with 0.1% Tween 20 in Tris-buffered saline (TBST) for 5 min each. The membranes were first blocked by incubating in a blocking solution (5% skim milk in 0.1% TBST) for 1 hr at RT and then treated with primary antibodies in the blocking solution overnight at 4 °C. The primary antibodies used were as follows: Ser262 (Thermo Fisher, 44-750G, 1:1,000), Ser404 (Thermo Fisher, 44-758G, 1:1,000), Ser396 (Abcam, ab109390, 1:1,000), AT8 (Thermo Fisher, MN1020, 1:1,000), Tau5 (Thermo Fisher, AHB0042, 1:2,000), GAPDH (Santa Cruz, sc-32233, 1:5,000), phospho-mTOR (Cell Signaling, 2971s, 1:1,000), mTOR (Cell Signaling, 2983s, 1:1,000), p62 (Cell Signaling, 5114s, 1:1,000), LC3B (Abcam, ab48394, 1:1,000).

The next day, the membranes were washed three times with TBST for 5 min each and incubated with horseradish peroxidase-conjugated secondary antibodies (1:5,000) against mouse or rabbit IgG in the blocking solution for 1 hr at RT. The membranes were washed five times with TBST 5 min each. Protein expression was detected using the West Pico PLUS Chemiluminescent Substrate (Thermo Fisher) and visualized using FUSION Solo 6S EDGE (Vilber Lourmat Sté, Collégien, France). The band intensity was quantified using ImageJ software (Bethesda, MD, USA).

### 2.8. Immunofluorescence

The harvested hearts were perfused with PBS and 4% PFA. The brains were excised and fixed in PFA overnight at 4 °C. Subsequently, they were sliced into 30 μm thick sections using a vibratome (VT-1200S; Leica Biosystems, Germany). The slice tissues were washed with PBS three times for 5 min each and incubated with a blocking solution (1% bovine serum albumin, 3% normal goat serum, and 0.4% Triton X-100 in PBS) for 1 hr at RT followed by incubation with anti-ionized calcium-binding adapter molecule 1 (Iba-1; Wako, 019–19741, 1:500) and anti-glial fibrillary acidic protein (GFAP; Dako, Z0334, 1:500) in blocking solution overnight at 4 °C. Then the tissue sections were washed with PBS three times for 5 min each and incubated with Alexa Fluor 488- or 594-conjugated secondary antibodies for 1 hr at RT in light-shielded conditions. The tissue slices were washed and mounted on glass slides. The images were captured using a K1-Fluo confocal microscope.

### 2.9. Statistical analysis

All data presented as mean ± standard error of the mean. The differences between the two groups were analyzed using the student's *t* test. One-way or Two-way analysis of variance followed by Dunnett’s multiple comparison post-hoc test was used for multiple group comparisons. For behavioral data obtained from the Morris water maze acquisition trials, mixed-effects model was used. Treatment group and training day were included as fixed effects, and mouse was specified as a random effect. The group × day interaction was incorporated into the model. Multiple comparisons were performed within each training day to compare treatment groups. All analyses were performed using GraphPad Prism (Version 8.0a; Graph Pad Software Inc, San Diego, CA, USA). A value of *p* < 0.05 was determined as statistically significant.

## 3. Results

### 3.1. Omipalisib reduces phosphorylated tau in SH-Tau cells

We treated SH-Tau cells with omipalisib to assess its cytotoxic effects. As omipalisib showed cytotoxicity at 10 nM but not at 1 nM, we analyzed the effects of omipalisib at concentrations below 1 nM (Fig 1B). To determine whether omipalisib reduced p-tau expression, SH-Tau cells were incubated with omipalisib (0.1, 0.3, or 1 nM) for 24 hr. At 1 nM, omipalisib significantly decreased the expression of Ser262 (*p* = 0.0139) and Ser404 (*p* = 0.0071), but not Tau5 in SH-Tau cells (Fig 1C-E). Additionally, the expression of p-tau was reduced by omipalisib treatment (1 nM) in a time-dependent manner (3 hr, *p* = 0.0074; 6 hr, *p* = 0.0057; 12 hr, *p* = 0.0003; 24 hr, *p* = 0.0006 for Ser262, 12 hr, *p* = 0.0063; 24 hr, *p* = 0.0007 for Ser404) (Fig 1F-H).

### 3.2. Omipalisib modulates autophagy-related protein via the mTOR pathway in SH-Tau cells

**Fig 2:**
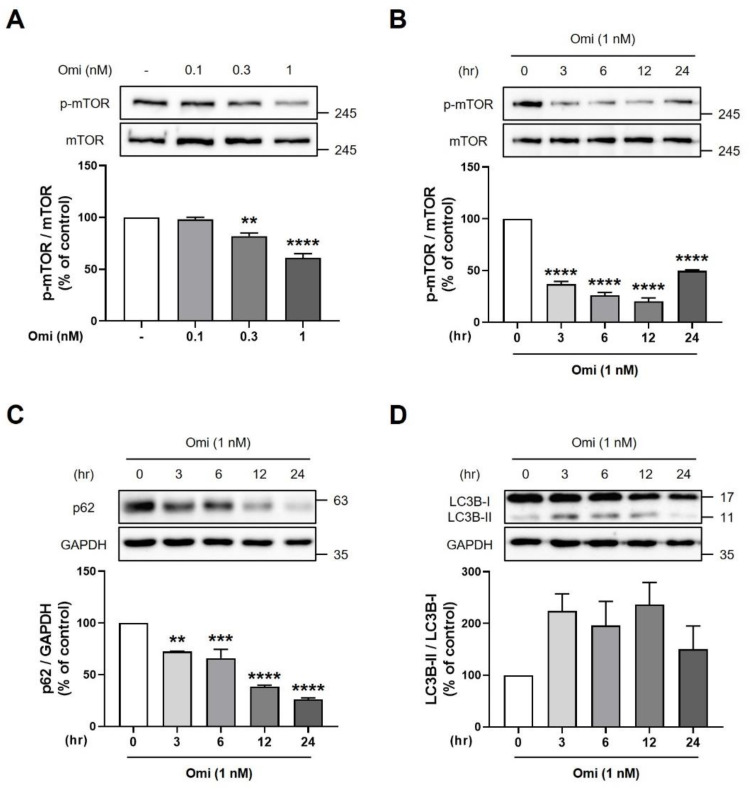
Effects of omipalisib on autophagy in SH-Tau cells. **(A and B)** Inhibition of phosphorylated mTOR by omipalisib in a dose- or time-dependent manner. **(A)** SH-Tau cells were treated with different concentrations (0.1–1 nM) of omipalisib for 24 hr. **(B)** SH-Tau cells were treated with omipalisib (1 nM) for different durations (0–24 hr). The levels of p-mTOR/mTOR in whole cell lysates were measured using western blot assay. **(C and D)** Changes of autophagy markers by omipalisib in a time-dependent manner. SH-Tau cells were treated with omipalisib (1 nM) for different durations (0–24 hr). The levels of p62 **(C)** and LC3B **(D)** in whole cell lysates were measured using western blot assay. GAPDH was used as an internal control. Statistical analysis was performed using one-way analysis of variance and Dunnett’s post hoc test. Data represent the mean ± SEM of three repeated experiments. *p < 0.05, **p < 0.01, ***p < 0.001, and ****p < 0.0001 compared to the control group.

Autophagy is a main pathway that clears accumulated p-tau [15]. Considering that omipalisib can inhibit the activation of mTOR, a factor that inhibits autophagy [10], we analyzed mTOR pathway-related autophagy factors. The levels of p-mTOR were significantly reduced following omipalisib treatment in a dose- (0.3 nM, *p* = 0.0044; 1 nM, *p* < 0.0001) and time- (3 hr, *p* < 0.0001; 6 hr, *p* < 0.0001; 12 hr, *p* < 0.0001; 24 hr, *p* < 0.0001) dependent manner (Fig 2A and B). To investigate the activation of the autophagy pathway by omipalisib (1 nM) treatment, the p62 and LC3B expression levels were analyzed. We observed a significant decrease in p62 levels following omipalisib treatment (3 hr, *p* = 0.0021; 6 hr, *p* = 0.0004; 12 hr, *p* < 0.0001; 24 hr, *p* < 0.0001) (Fig 2C). Although LC3B-II/LC3B-I ratios showed a trend towards an increase, these changes were not significant (3 hr, *p* = 0.1237; 6 hr, *p* = 0.2776; 12 hr, *p* = 0.0857; 24 hr, *p* = 0.7543) (Fig 2D). Furthermore, omipalisib significantly reduced Ser262 levels (*p* = 0.0207), whereas co-treatment with the autophagy inhibitor 3-MA blocked this reduction (*p* = 0.0007) (S4 Fig). These data indicated that omipalisib decreased the levels of accumulated p-tau by modulating ALP signaling through mTOR inhibition.

### 3.3. Omipalisib decreases phosphorylated tau levels in the brain of PS19 mice

**Fig 3:**
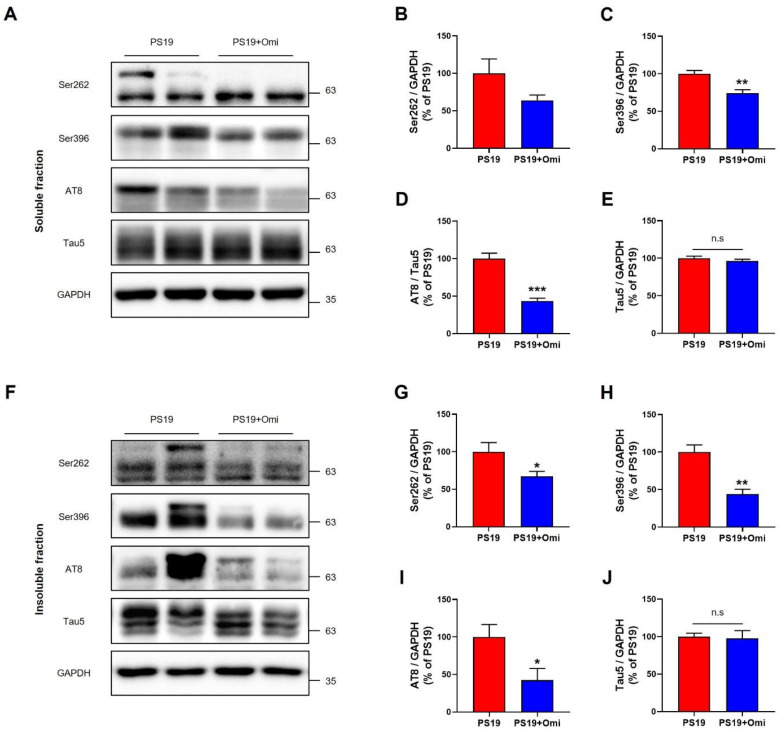
Effects of omipalisib on the overexpression of phosphorylated tau in PS19 mice. Brain lysates were separated into soluble and insoluble fractions in RIPA buffer. **(A)** Western blot band of the RIPA-soluble fraction. **(B-E)** The quantification of Ser262 **(B)**, Ser396 **(C)**, AT8 **(D)**, and Tau5 **(E)** were normalized to GAPDH (*n* = 5 per group). P-tau levels were measured in PS19 mice only, as the analysis focused on human tau expressed in the PS19 mice. **(F)** Western blot band of the RIPA-insoluble fraction. **(G-J)** The quantification of Ser262 **(G)**, Ser396 **(H)**, AT8 **(I)**, and Tau5 **(J)** were normalized to GAPDH (*n* = 5 per group). Statistical analysis was performed using the student’s *t* test. Data represent the mean ± SEM. *p < 0.05, **p < 0.01, and ***p < 0.001 compared to the PS19 group. n.s, nonsignificant.

**Fig 4:**
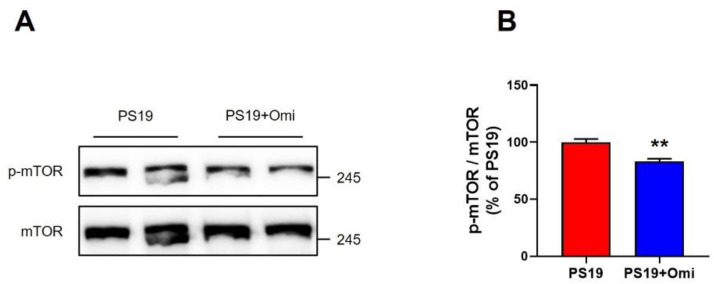
Effects of omipalisib on mTOR signaling in PS19 mice. **(A)** Western blot band of p-mTOR and mTOR. **(B)** The quantification of p-mTOR was normalized to mTOR (*n* = 5 per group). Statistical analysis was performed using the student’s *t* test. Data represent the mean ± SEM. **p < 0.01 compared to the PS19 group.

To examine whether omipalisib reduced p-tau levels *in vivo*, we used PS19 mice, a well-known animal model of tauopathy. PS19 mice were administered omipalisib (1 mg/kg/day) for 2 months. We separated the brain proteins into soluble and insoluble fractions and measured the levels of p-tau in each fraction. Omipalisib treatment significantly reduced the levels of soluble p-tau at Ser396 (*p* = 0.0041) and AT8 (*p* = 0.0001), whereas soluble p-tau at Ser262 did not show a significant reduction (*p* = 0.1175) (Fig 3A-D). Moreover, levels of insoluble p-tau (Ser262, *p* = 0.0492; Ser396, *p* = 0.0055; AT8, *p* = 0.0383) were also markedly reduced in the brains of omipalisib-treated PS19 mice (Fig 3F-I). It appears that the number of Ser396-positive cells was reduced in omipalisib-treated PS19 mice similar to the WB data for Ser396 (S5 Fig). Expression of Tau5 did not change in both soluble and insoluble form (Fig 3E and J). We investigated whether the effect of omipalisib on p-tau degradation was mediated by mTOR signaling. As expected, p-mTOR expression of omipalisib-treated PS19 mice was significantly lower than those of vehicle-treated PS19 mice (*p* = 0.0017) (Fig 4).

Next, we stained the brains for Iba-1 (a microglial marker) and GFAP (an astrocyte marker) to determine changes in neuroinflammation, which is another hallmark of tauopathy. Notably, omipalisib treatment did not alleviate astrocyte and microglia activation in PS19 mice (S6 Fig).

### 3.4. Omipalisib ameliorates memory dysfunction in PS19 mice

**Fig 5:**
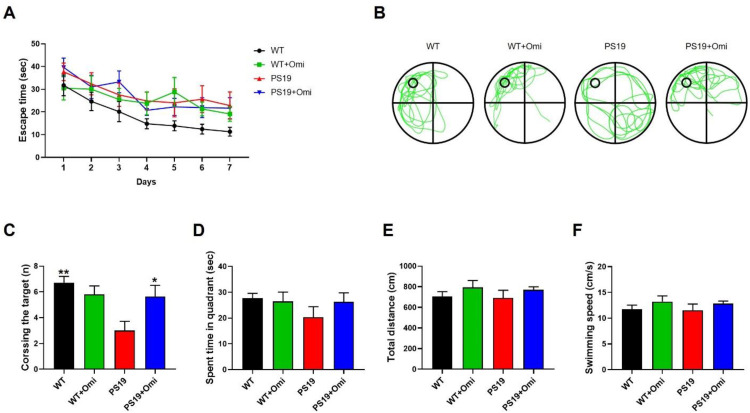
Effects of omipalisib on memory dysfunction in PS19 mice using the Morris water maze test. **(A)** Escape time of animals over a 7-day training period (vehicle-treated WT, *n* = 10; omipalisib-treated WT, *n* = 10; vehicle-treated PS19, *n* = 9; omipalisib-treated PS19, *n* = 11). **(B)** Representative swimming paths on day 8. **(C-F)** On probe trial day 8, the number of target crossings **(C)**, time spent in the quadrant **(D)**, total distance covered **(E)**, and swimming speed **(F)** were measured and analyzed. Statistical analysis was performed using one-way or two-way analysis of variance and Dunnett’s post hoc test. Morris water maze acquisition data were analyzed using a mixed-effects model. Data represent the mean ± SEM. *p < 0.05 and **p < 0.01 compared to vehicle-treated PS19 mice.

Finally, to determine whether the reduction in p-tau expression by omipalisib influences learning and memory, the MWM test was performed. The escape time (s) of omipalisib-treated PS19 mice was shorter than that of the PS19 mice (Fig 5A). The representative navigation paths on day 8 of training showed that PS19 mice treated with omipalisib displayed a navigation pattern similar to that of WT mice (Fig 5B). Moreover, omipalisib-treated PS19 mice passed the platform more times than the PS19 mice (Fig 5C), and the time spent in the quadrant where the platform was located was longer (Fig 5D), without a decrease in motor function (Fig 5E and F). It should also be noted that the partial behavioral rescue observed in our study may reflect the complex molecular signatures and diverse pathological pathways involved in tauopathy, suggesting that multiple mechanisms likely contribute to functional outcomes in tauopathy models [16].

## 4. Discussion

The accumulation of p-tau plays a crucial role in the progression of primary and secondary tauopathies. Tau protein is generally involved in stabilizing microtubules in neurons but becomes hyperphosphorylated in tauopathies. This interferes with normal cellular functions, leading to cognitive impairment, synaptic dysfunction, and neuronal loss [17]. Therefore, the removal of abnormally accumulated tau is crucial for the prevention of disease progression and to restore normal cellular function. Currently, several studies are investigating therapeutic approaches to inhibit tau aggregation or improve p-tau clearance.

Autophagy is an essential cellular mechanism that degrades and recycles abnormal proteins and damaged organelles to maintain cellular homeostasis [18]. Given that autophagy is a potential mechanism for eliminating pathological tau accumulation, a hallmark of tauopathy, several studies have focused on regulating mTOR, a major inhibitory signaling pathway of autophagy, through pharmacological agents [19]. Pharmacological approaches provide another strategy for enhancing autophagy. Rapamycin, a well-known mTOR inhibitor, reduces aggregated or phosphorylated tau, resulting in amelioration of gliosis, neuronal loss, synaptotoxicity, and cognitive impairment in mouse models of tauopathy [8,20–22]. In addition, preclinical studies have evaluated the effect of several autophagy inducers, including mTOR-dependent (methylene blue and curcumin) [23,24] and mTOR-independent (metformin, trehalose, and lithium) [25–27], on p-tau expression. NVP-BEZ235, a dual PI3K/mTOR inhibitor, has also been shown to decrease p-tau levels through the autophagy pathway [28]. These findings suggest that autophagy modulation through the inhibition of mTOR signaling is a potential therapeutic approach for tauopathy. This reduction in p-tau aligns with established frameworks in which targeting tau-related kinase pathways mitigate pathological tau accumulation [29]. Furthermore, such regulation is important for maintaining cellular proteostasis, as abnormal tau modifications can disrupt broader protein homeostasis networks [30].

In our study, we hypothesized that omipalisib, a PI3K/mTOR inhibitor, could remove accumulated tau and inhibit memory decline by activating autophagy. Omipalisib promotes autophagy by decreasing PI3K/mTOR signaling, resulting in exacerbated cell death in cancer cell lines [10,12]. Furthermore, clinical trials have demonstrated acceptable tolerability of omipalisib as a PI3K/mTOR inhibitor in patients with advanced solid tumors or idiopathic pulmonary fibrosis [9,31]. Pharmacokinetic study has shown that omipalisib is detectable in the brain after oral administration at 10 mg/kg in normal mice, suggesting partial BBB permeability under normal conditions [32]. Additionally, omipalisib has a logP value of approximately 3.2, one hydrogen bond donor, and seven hydrogen bond acceptors, properties that fall within the physicochemical range generally associated with BBB permeability. These findings indicate that omipalisib has the potential to penetrate the BBB, but additional confirmation would be valuable with the condition used in this study. In this study, we observed that omipalisib reduced p-mTOR expression in a time- and dose-dependent manner and dramatically altered the expression of the autophagic markers (p62 and LC3B). Additionally, omipalisib decreased p-tau expression without reducing total tau expression in both *in vitro* and *in vivo* tau models. Although omipalisib has the potential to influence the PI3K-Akt-glycogen synthase kinase 3β (GSK3β) pathway, p-GSK3β (Ser9) levels were not significantly altered (S7 Fig). These results suggest that the reduction of p-tau by omipalisib may be associated with changes in mTOR-mediated autophagy signaling. Further studies are required to thoroughly and extensively evaluate the mechanisms of omipalisib action.

While our findings support a potential therapeutic role of omipalisib in the tau pathology, several methodological limitations need to be addressed in future work. Although 3-MA was used to assess whether omipalisib-induced effects are modulated by autophagy, autophagic flux was not directly confirmed using lysosomal inhibitors or genetic approaches, and additional studies are required to further clarify this mechanism. In addition, possible involvement of autophagy in vivo was inferred from upstream signaling changes, as downstream autophagy markers such as LC3-II and p62 were not directly assessed in brain tissue. Therefore, our findings indicate a potential association between omipalisib treatment and changes in autophagy markers, but additional experiments are required to fully demonstrate autophagic flux. The second limitation of our study is that the in vitro and in vivo systems express different N-terminal tau isoforms (0N4R vs 1N4R). Although N-terminal differences are known to affect tau aggregation kinetics [33], both models express 4R tau harboring P301X mutations, which are known to impair microtubule binding and promote aggregation [34,35]. Thus, we focused on directional consistency of the main phenotypes (e.g., reduction in p-tau and enhancement of autophagy) across models. Future studies using matched isoform/mutation combinations would strengthen translational interpretation.

In conclusion, the results of this study show that omipalisib decreases excessive p-tau expression and mitigates cognitive impairment in both *in vitro* and *in vivo* tauopathy models. These therapeutic effects of omipalisib appear to involve modulation of the mTOR-mediated autophagy signals. Although autophagic flux was not directly measured, our findings suggest a potential association between omipalisib treatment and pathways involved in tau clearance. Overall, this study highlights the potential of omipalisib as a candidate therapeutic agent for tau-related neurodegenerative disorders.

## Supporting information

S1 Fig Pilot tolerability assessment in mice. **(A)** Humane endpoint-free survival of mice treated with omipalisib at 1 mg/kg and 3 mg/kg for 7 days. **(B)** Body weight changes in mice treated with 1 mg/kg omipalisib for 7 days. Data represent the mean ± SEM.
(DOCX)

S2 Fig Schematics illustrating the protocol of omipalisib treatment in PS19 mice. i.p: Intraperitoneal injection; MWM: Morris water maze.
(DOCX)

S3 Fig Body weight changes for 2 months of omipalisib treatment in WT and PS19 mice. Data represent the mean ± SEM.
(DOCX)

S4 Fig Co-treatment with 3-MA blocks omipalisib-induced reduction of p62 and phosphorylated tau. **(A)** Western blot band of p62, Ser262, and GAPDH. **(B)** The quantification of p62 was normalized to GAPDH. **(C)** The quantification of Ser262 was normalized to GAPDH. Statistical analysis was performed using one-way analysis of variance and Dunnett’s post hoc test. Data represent the mean ± SEM. *p < 0.05 compared to the control group. ###*p* < 0.001 compared to the omipalisib group.
(DOCX)

S5 Fig Effect of omipalisib on phosphorylated tau in PS19 mice. Brain sections were stained with anti-Ser396 antibody. Scale bar: 100 μm.
(DOCX)

S6 Fig Effect of omipalisib on gliosis. Brain sections were stained with anti-GFAP and Iba-1 antibody. Scale bar: 100 μm.
(DOCX)

S7 Fig Effects of omipalisib on GSK3β in SH-Tau cells. **(A)** Western blot band of p-GSK3β and GSK3β. **(B)** The quantification of p-GSK3β was normalized to GSK3β. Statistical analysis was performed using one-way analysis of variance and Dunnett’s post hoc test. Data represent the mean ± SEM of three repeated experiments.
(DOCX)

## Abbreviation:

ALP: autophagy-lysosome pathway
DMSO: dimethyl sulfoxide
FBS: fetal bovine serum
GFAP: glial fibrillary acidic protein
MWM: Morris water maze test
mTOR: mammalian target of rapamycin
PBS: phosphate-buffered saline
PFA: paraformaldehyde
PI3K: phosphoinositide 3-kinase
PS19: tau P301S mutant mice
P/S: penicillin-streptomycin
p-tau: hyperphosphorylated tau
RIPA: radioimmunoprecipitation assay
RPMI: Roswell Park Memorial Institute media
RT: room temperature
SDS: sodium dodecyl sulfate
SH-Tau: P301L mutant tau-expressing SH-SY5Y cells
TBST: tris-buffered saline
WST-1: water-soluble tetrazolium
WT: wild-type
