# Supplementary figures and images for: Omipalisib reduces hyperphosphorylated tau protein by modulating mTOR-autophagy pathway
Source: PLoS One. 2026 Jun 23;21(6):e0352120. doi: 10.1371/journal.pone.0352120 (PMC13289877; doi:10.1371/journal.pone.0352120)

**Supporting information 1**


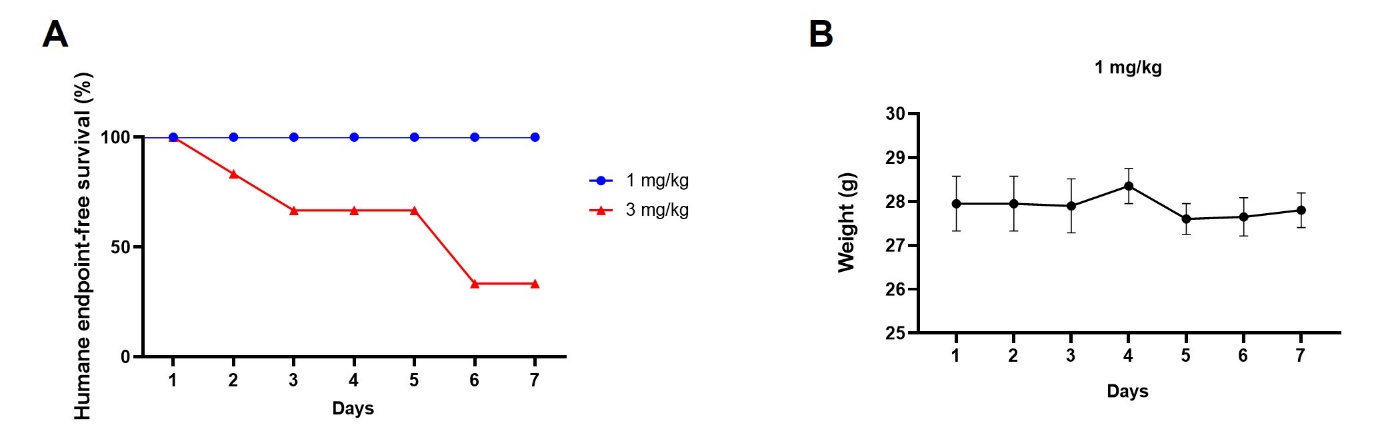

Supplement: S1 Fig — (A) Humane endpoint-free survival of mice treated with omipalisib at 1 mg/kg and 3 mg/kg for 7 days. (B) Body weight changes in mice treated with 1 mg/kg omipalisib for 7 days. Data represent the mean ± SEM. (DOCX) [file pone.0352120.s001.docx]

**Supporting information 2**


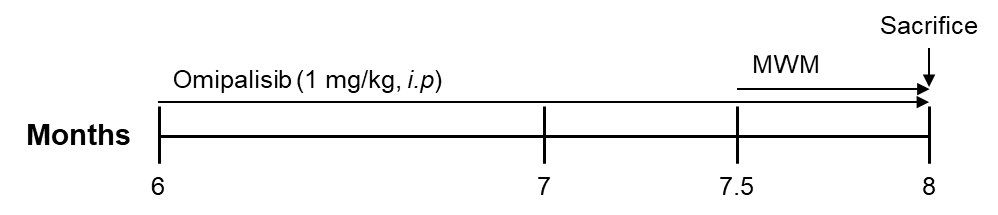

Supplement: S2 Fig — i.p: Intraperitoneal injection; MWM: Morris water maze. (DOCX) [file pone.0352120.s002.docx]

**Supporting information 3**


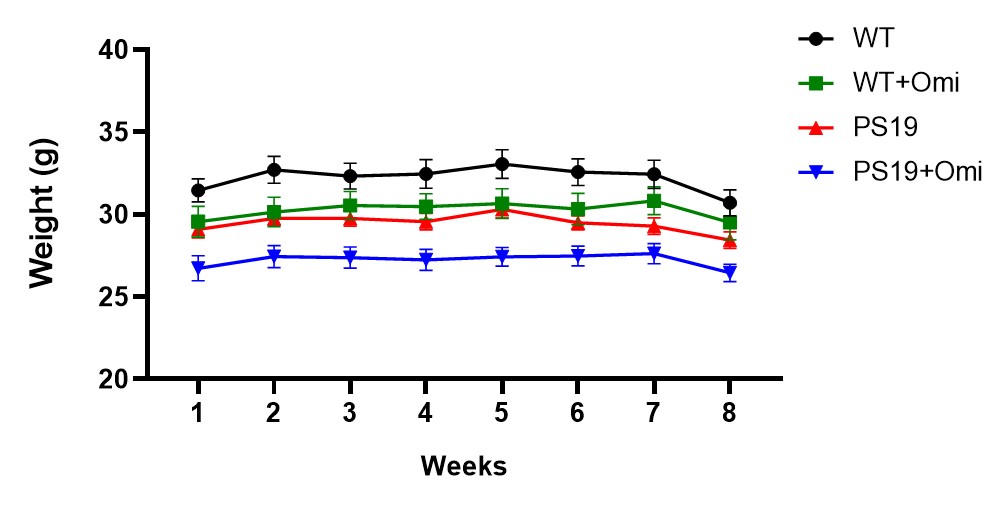

Supplement: S3 Fig — Data represent the mean ± SEM. (DOCX) [file pone.0352120.s003.docx]

**Supporting information 4**


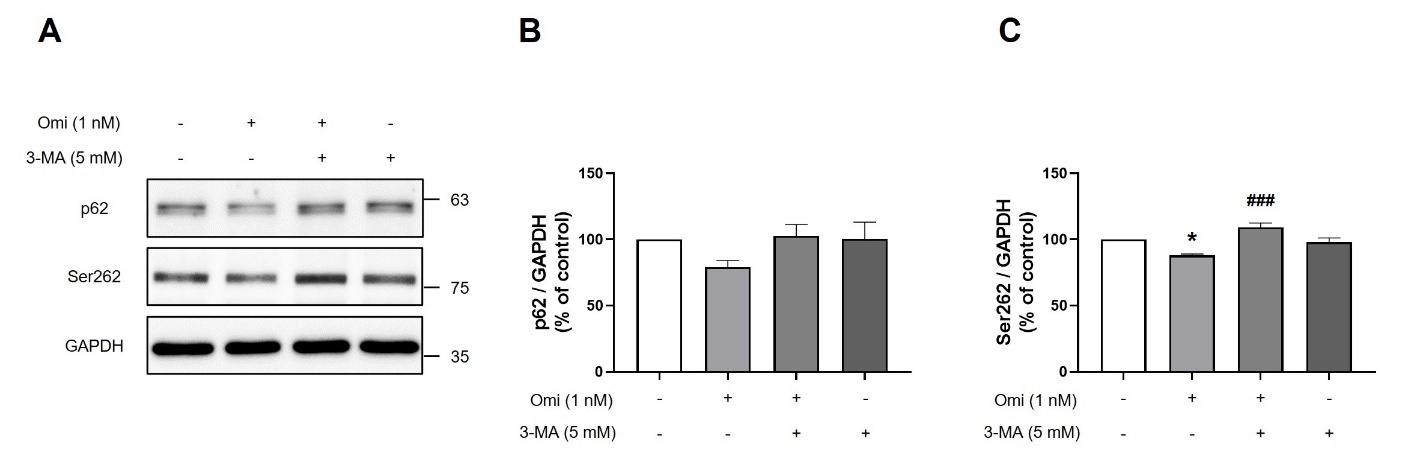

Supplement: S4 Fig — (A) Western blot band of p62, Ser262, and GAPDH. (B) The quantification of p62 was normalized to GAPDH. (C) The quantification of Ser262 was normalized to GAPDH. Statistical analysis was performed using one-way analysis of variance and Dunnett’s post hoc test. Data represent the mean ± SEM. *p < 0.05 compared to the control group. ###p < 0.001 compared to the omipalisib group. (DOCX) [file pone.0352120.s004.docx]

**Supporting information 5**


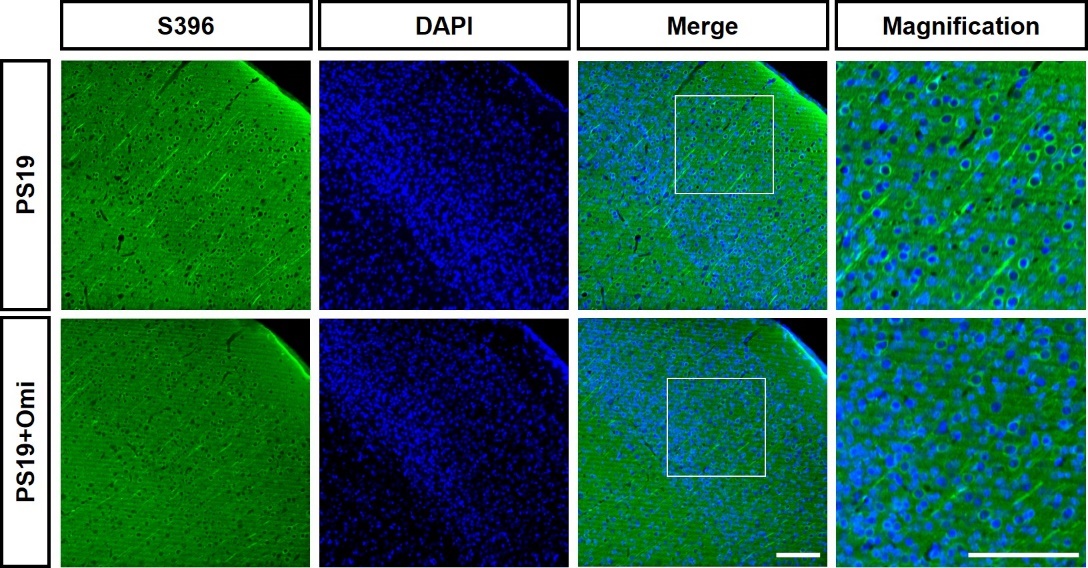

Supplement: S5 Fig — Brain sections were stained with anti-Ser396 antibody. Scale bar: 100 μm. (DOCX) [file pone.0352120.s005.docx]

**Supporting information 6**


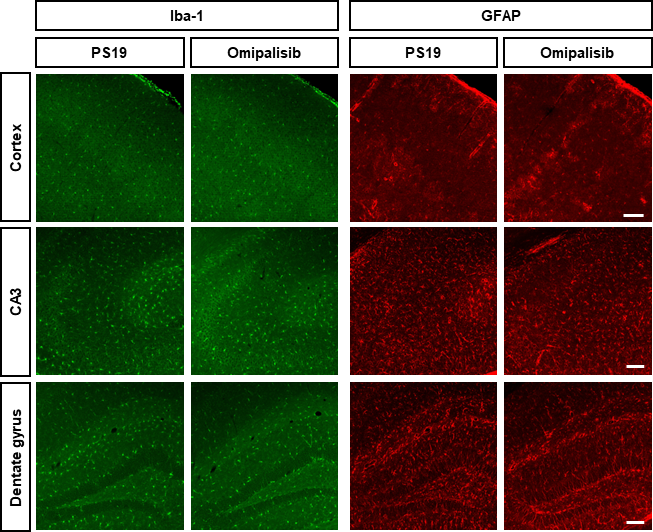

Supplement: S6 Fig — Brain sections were stained with anti-GFAP and Iba-1 antibody. Scale bar: 100 μm. (DOCX) [file pone.0352120.s006.docx]

**Supporting information 7**


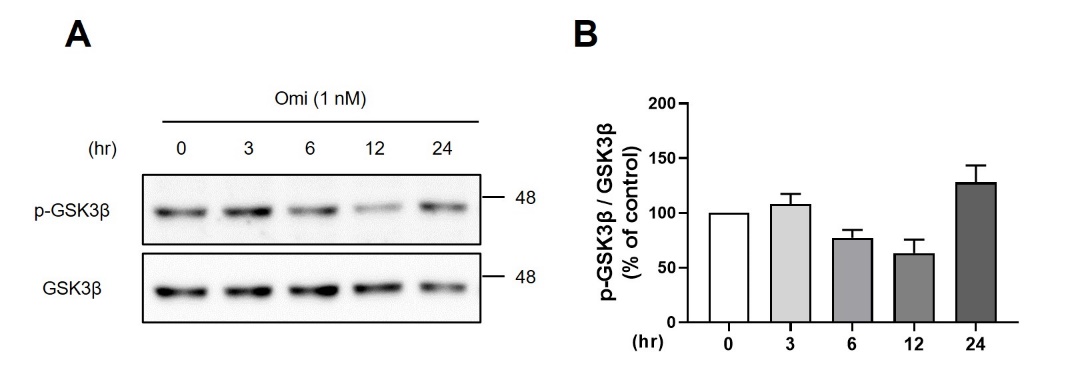

Supplement: S7 Fig — (A) Western blot band of p-GSK3β and GSK3β. (B) The quantification of p-GSK3β was normalized to GSK3β. Statistical analysis was performed using one-way analysis of variance and Dunnett’s post hoc test. Data represent the mean ± SEM of three repeated experiments. (DOCX) [file pone.0352120.s007.docx]
