# Supplementary material for: Omipalisib reduces hyperphosphorylated tau protein by modulating mTOR-autophagy pathway
Source: PLoS One. 2026 Jun 23;21(6):e0352120. doi: 10.1371/journal.pone.0352120 (PMC13289877; doi:10.1371/journal.pone.0352120)
Supplement: Attachment — Submitted filename: Response to Reviewers.docx [file pone.0352120.s008.docx]

**Dear Editor and Reviewers,**

We appreciate your kind and wise comments, the opportunity to revise our work, and your review of this work. We have responded to the comments as follows and revised the manuscript after carefully considering your suggestions. In the revised manuscript, the changed parts were written in blue font.

**----------------------------------------------------------------------------------------------------------------**

**Regarding the Reviewer #1’s comments**

**Question 1:** Autophagy Not Directly Proven - LC3 and p62 levels are shown, but autophagic flux is not assessed. Recommend adding bafilomycin/lysosomal inhibitor experiments or using Atg5/Atg7 knockdown to confirm flux. If not feasible, tone down conclusions and acknowledge this limitation.

**Response 1:** We appreciate the reviewer’s insightful comment regarding autophagy flux. We performed an additional experiment using 3-methyladenin (3-MA), an early-stage autophagy inhibitor, to pharmacologically block autophagy. As in the revised figure, the reduction of p-tau by omipalisib was abolished when cells were co-treated with 3-MA, suggesting that omipalisib reduces p-tau levels through activation of autophagy. We added the results in the revised manuscripts as follows:

- Page 10, line 21 in the results

Furthermore, omipalisib significantly reduced Ser262 levels (*p* = 0.0207), whereas co-treatment with the autophagy inhibitor 3-MA blocked this reduction (*p* = 0.0007). (Fig. S3).

**Fig. S3** Co-treatment with 3-MA blocks omipalisib-induced reduction of p62 and p-tau. (A) Western blot band of p62, Ser262, and GAPDH. (B) The quantification of p62 was normalized to GAPDH. (C) The quantification of Ser262 was normalized to GAPDH. Statistical analysis was performed using one-way analysis of variance and Dunnett’s post hoc test. Data represent the mean ± SEM. **p* < 0.05 compared to the control group. ###*p* < 0.001 compared to the omipalisib group.

Although we performed an additional experiment using 3-MA, we agree with the reviewer that direct confirmation of autophagic flux would further strengthen our conclusion. Therefore, we have discussed this as a limitation in the revised manuscripts as follows:

- Page 14, line 1 in the discussion

Although we assessed autophagy modulation using 3-MA, autophagic flux was not directly confirmed with lysosomal inhibitors or genetic approaches. Thus, our findings indicate that omipalisib may reduce p-tau via autophagy activation, but additional experiments are required to fully demonstrate autophagic flux.

**Question 2:** Mechanistic Specificity - Omipalisib is inaccurately described as a "selective mTOR inhibitor" — it's a dual PI3K/mTOR inhibitor. Please revise all text accordingly and discuss implications (e.g., PI3K→Akt→GSK3β feedback on tau kinases).

**Response 2:** We thank the reviewer for pointing out a potential lack of clarity in our study. We have carefully changed description about omipalisib in the revised manuscripts as follows:

- Page 3, line 6 in the abstract

Omipalisib, a dual phosphatidylinositol 3-kinase/mammalian target of rapamycin (PI3K/mTOR) inhibitor, was explored in a phase I clinical trial involving solid tumors and lymphoma.

- Page 13, line 11 in the discussion

In our study, we hypothesized that omipalisib, a PI3K/mTOR inhibitor, could remove accumulated tau and inhibit memory decline by activating autophagy.

We agree with the reviewer that omipalis, as a PI3K/mTOR inhibitor, could influence the PI3K-Akt-GSK3β signaling. However, under our experimental conditions, we did not observe GSK3β activation (decreased Ser9 level) by omipalisib treatment (Additional figure. 1). And GSK3β can be regulated not only by PI3K/Akt signaling but also through multiple alternative mechanisms [Cell. 2017 Apr 20;169(3):381-405.]. Based on our data, we interpret that omipalisib reduced p-Tau levels through mTOR-mediated autophagy without detectable changes in GSK3β. We have carefully discussed this point in the revised manuscript as follows:

- Page 13, line 16 in the discussion

Additionally, omipalisib decreased p-tau expression without reducing total tau expression in both *in vitro* and *in vivo* tau models. Although omipalisib could theoretically affect the PI3K-Akt-glycogen synthase kinase 3β (GSK3β) pathway, we did not observe significant changes in p-GSK3β (Ser9) (data not shown). These results suggest that omipalisib reduced p-tau levels through mTOR-mediated autophagy in a tau-overexpressing environment.

**Additional Figure. 1** Effects of omipalisib on GSK3β in SH-Tau cells. (A) Western blot band of p-GSK3β and GSK3β. (B) The quantification of p-GSK3β was normalized to GSK3β. Statistical analysis was performed using one-way analysis of variance and Dunnett’s post hoc test. Data represent the mean ± SEM of three repeated experiments.

**Question 3:** In Vivo Mechanism Confirmation Needed - No evidence of mTOR inhibition or autophagy activation in mouse brain. Recommend measuring p-mTOR, LC3-II, or p62 in PS19 mouse brain lysates.

**Response 3:** We thank the reviewer for this comment. According to the reviewer’s comment, we have analyzed expression of phosphorylated mTOR (p-mTOR) in the brain. As expected, the levels of p-mTOR/mTOR was decreased after omipalisib treatment in PS19 mice. We have added this data in the Figure 4 of revised manuscript as follows:

- Page 11, line 14 in the results

We investigated whether the effect of omipalisib on p-tau degradation was mediated by mTOR signaling. As expected, p-mTOR expression of omipalisib-treated PS19 mice was significantly lower than those of vehicle-treated PS19 mice (*p* = 0.0017) (Fig. 4).

**Fig. 4** Effects of omipalisib on mTOR signaling in PS19 mice. (A) Western blot band of p-mTOR and mTOR. (B) The quantification of p-mTOR was normalized to mTOR (*n* = 5 per group). Statistical analysis was performed using the student’s t test. Data represent the mean ± SEM. ***p* < 0.01 compared to the PS19 group.

**Question 4:** Histological Validation Missing - Western blot shows tau reduction; immunohistochemistry for p-tau (e.g., AT8), and neuroinflammation (GFAP, Iba1) would strengthen the case.

**Response 4:** We would like to thank the reviewer for this comment. According to the reviewer's comment, we have performed immunostaining of p-tau using Ser396 antibody. As shown in supplementary figure 4, positive cells of p-tau were decreased after omipalisib treatment in PS19 mice. We couldn’t observe the change of neuroinflammation (GFAP and Iba-1) in the brains of mice (Fig. S5). We have added this data in the supplementary figure 4 of revised manuscript as follows:

- Page 11, line 11 in the results

It appears that the number of Ser396-positive cells was reduced in omipalisib-treated PS19 mice similar to the WB data for Ser396 (Fig. S4).

**Fig. S4** Effect of omipalisib on phosphorylated tau (p-tau) in PS19 mice. Brain sections were stained with anti-Ser396 antibody. Scale bar: 100 μm.

**Question 5:** Clarify Behavioral Analysis - Use repeated-measures ANOVA for MWM training.

**Response 5:** We thank the reviewer for the comment. According to the reviewer’s comment, we have clarified the statistical analysis. Figure 4A was analyzed using two-way ANOVA and Figures 5C–F were analyzed using one-way ANOVA. We have carefully added this changed analysis in the revised manuscript and revised figure as follows:

- Page 9, line 15 in the methods

One-way or Two-way analysis of variance followed by Dunnett’s multiple comparison post-hoc test was used for multiple group comparisons.

- Page 6, line 5 in the figure 5 caption

Statistical analysis was performed using one-way or two-way analysis of variance and Dunnett’s post hoc test. Data represent the mean ± SEM.

**Question 6:** Confirm motor performance was unaffected (distance, speed) and report blinding.

**Response 6:** Thank you for the reviewer’s comment. We monitored the total distance and swimming speed of mice during Morris water-maze probe test and found no significant differences (Fig. 5). Also, the experiment was conducted under blinded conditions, and we have carefully added a sentence describing about the blinding procedure in the revised manuscripts as follows:

- Page 6, line 20 in the methods

Mice were assigned to experimental groups using a block randomization method. To minimize bias, all behavioral testing and quantitative analyses were conducted under blinded conditions.

**Question 7:** Safety Observations in Mice - Report any weight changes, health issues, or behavioral effects of chronic Omipalisib treatment.

**Response 7:** Thank you for the reviewer’s thoughtful comment. We monitored body weight once a week during the 2 months of omipalisib treatment and found no significant differences between vehicle-treated groups and omipalisib-treated groups. No visible signs of health problems were observed. Furthermore, the swimming speed in the probe test was not reduced in omipalisib-treated groups compared with vehicle-treated groups. Therefore, we believe that the treatment with omipalisib at 1 mg/kg for 2 months did not induce adverse effects. According to the reviewer’s comment, we carefully added the weight result in the revised manuscripts as follows:

- Page 6, line 17 in the methods

Body weight of the mice was measured weekly throughout the treatment period to monitor any potential changes (Fig. S2).

**Fig. S2** Body weight changes for 2 months of omipalisib treatment in WT and PS19 mice. Data represent the mean ± SEM.

**Question 8:** Improve Discussion - Compare to other autophagy inducers (rapamycin, trehalose, metformin, BEZ235). Acknowledge Omipalisib’s systemic toxicity risks and translational challenges.

**Response 8:** We appreciate the comment. Since omipalisib is a dual PI3K/mTOR inhibitor originally developed as an anticancer agent, we referred to the published article [Med Sci Monit. 2020 Aug 17:26:e927106] to select the appropriate omipalisib dosage and minimize potential systemic toxicity. Following that result, we performed a pilot study testing 1 and 3 mg/kg of omipalisib and monitored survival and body weight. In our protocol, 3 of 7 mice treated with 3 mg/kg of omipalisib died, whereas there was no dead mouse in the 1 mg/kg group and body weight in this group also didn’t significantly change (Additional figure. 2). Based on these observations, we selected 1 mg/kg of omipalisib for our main experiment.

**Additional Figure. 2** Effects of omipalisib on survival and body weight in mice. (A) Survival of mice treated with omipalisib at 1 mg/kg and 3 mg/kg for 7 days. (B) Body weight changes in mice treated with 1 mg/kg omipalisib for 7 days. Data represent the mean ± SEM.

According to the reviewer’s comment, we have carefully added information for other autophagy inducers in the revised manuscripts as follows:

- Page 13, line 2 in the discussion

In addition, preclinical studies have evaluated the effect of several autophagy inducers, including mTOR-dependent (methylene blue and curcumin) (22, 23) and mTOR-independent (metformin, trehalose, and lithium) (24-26), on p-tau expression. NVP-BEZ235, a dual PI3K/mTOR inhibitor, has also been shown to decrease p-tau levels through the autophagy pathway (27).

**Question 9:** Title: Rephrase to “...via mTOR inhibition” (mTOR inhibits autophagy, not activates it).

**Response 9:** We thank the reviewer for pointing out a potential lack of clarity in our study. According to the reviewer’s comment, we have carefully edited that title to provide the reader with a clearer understanding of paper in the revised manuscripts as follows:

- Page 1, line 1 in the manuscript

Omipalisib reduces hyperphosphorylated tau protein by activating autophagy through mTOR inhibition

**Question 10:** Clarify “ALS” (autophagy–lysosome system) to avoid confusion with amyotrophic lateral sclerosis.

**Response 10:** We appreciate the reviewer’s comment. To avoid confusion, all occurrences of “ALS” have been replaced with “autophagy-lysosome pathway (ALP)” throughout the manuscript.

- Page 10, line 23 in the results

These data indicated that omipalisib decreased the levels of accumulated p-tau by activating ALP through mTOR inhibition.

**Question 11:** Western blots: Show representative images in figures or supplementary files.

**Response 11:** Thank you for the reviewer’s comment. We have clearly indicated the representative blots corresponding to those shown in the main figure. The selected blots are highlighted with boxes in the supporting information (Western blot images).

**Question 12:** Reference: Add more on dual inhibitors in tauopathy (e.g., BEZ235).

**Response 12:** Thank you for the reviewer’s comment. According to the reviewer’s comment, we have carefully added reference for BEZ235 in the revised manuscripts as follows:

- Page 13, line 5 in the discussion

NVP-BEZ235, a dual PI3K/mTOR inhibitor, has also been shown to decrease p-tau levels through the autophagy pathway (27).

**Question 13:** Methods: Clarify drug formulation, injection route, randomization/blinding, sex of mice.

**Response 13:** We appreciate the reviewer’s comments. In this study, only male mice were used, and omipalisib was administered intraperitoneally after being dissolved in 10% DMSO and 5% Tween-80. We have carefully added detailed information in the revised manuscripts as follows:

- Page 5, line 14 in the materials

Omipalisib (C_25_H_17_F_2_N_5_O_3_S, HY-10297, > 99% purity, Fig. 1A) was purchased from MedChemExpress (Monmouth Junction, NJ, USA).

- Page 6, line 20 in the methods

Mice were assigned to experimental groups using a block randomization method. To minimize bias, all behavioral testing and quantitative analyses were conducted under blinded conditions.

**Question 14:** Statistical details: Provide exact p-values where possible; confirm correction for multiple comparisons.

**Response 14:** Thank you for your kind comment.Exact p-values have been carefully added in the revised manuscript as follows:

- Page 10, line 3 in the results

To determine whether omipalisib reduced p-tau expression, SH-Tau cells were incubated with omipalisib (0.1, 0.3, or 1 nM) for 24 hr. At 1 nM, omipalisib significantly decreased the expression of Ser262 (*p* = 0.0139) and Ser404 (*p* = 0.0071), but not Tau5 in SH-Tau cells (Fig. 1C-E). Additionally, the expression of p-tau was reduced by omipalisib treatment (1 nM) in a time-dependent manner (3 hr, *p* =0.0074; 6 hr, *p* = 0.0057; 12 hr, *p* = 0.0003; 24 hr, *p* = 0.0006 for Ser262, 12 hr, *p* = 0.0063; 24 hr, *p* = 0.0007 for Ser404) (Fig. 1F-H).

- Page 10, line 14 in the results

The levels of p-mTOR were significantly reduced following omipalisib treatment in a dose- (1 nM, *p* = 0.0148) and time- (3 hr, *p* < 0.0001; 6 hr, *p* < 0.0001; 12 hr, *p* <0.0001; 24 hr, *p* < 0.0001) dependent manner (Fig. 2A and B).

- Page 10, line 18 in the results

We observed a decrease in p62 levels and an increase in LC3B-II/LC3B-I ratios after 3 hr of omipalisib treatment (3 hr, *p* = 0.0021; 6 hr, *p* = 0.0004; 12 hr, *p* < 0.0001 ; 24 hr, *p* < 0.0001 for p62, (3 hr, *p* = 0.1237; 6 hr, *p* = 0.2776; 12 hr, *p* = 0.0857; 24 hr, *p* = 0.7543 for LC3B-II) (Fig. 2C and D).

- Page 11, line 8 in the results

Omipalisib treatment reduced the levels of soluble p-tau (Ser262, *p* = 0.1175; Ser396, *p* = 0.0041; AT8, *p* = 0.0001) (Fig. 3A-D). Moreover, levels of insoluble p-tau (Ser262, *p* = 0.0492; Ser396, *p* = 0.0055; AT8, *p* = 0.0383) were also markedly reduced in the brains of omipalisib-treated PS19 mice (Fig. 3F-I).

- Page 11, line 14 in the results

We investigated whether the effect of omipalisib on p-tau degradation was mediated by mTOR signaling. As expected, p-mTOR expression of omipalisib-treated PS19 mice was significantly lower than those of vehicle-treated PS19 mice (*p* = 0.0017) (Fig. 4).

**Question 15:** Language edits: Fix typos (e.g., “preformed” → “performed”), and check for grammar and clarity.

**Response 15:** Thank you for the reviewer’s comment. We have fixed the word in the revised figure as follows:

- Page 1, line 1 in the supplementary figure 1 caption

Schematics illustrating the protocol of omipalisib treatment in PS19 mice. i.p: Intraperitoneal injection; MWM: Morris water maze.

**Regarding the Reviewer #2’s comments**

**Question 1:** In Figure 3 (tau Western blots) and Supplementary Fig. S2 (Neuroinflammation data), WT groups are missing, despite being described in the Methods section (four groups: WT+vehicle, WT+Omipalisib, PS19+vehicle, PS19+Omipalisib). The absence of WT controls significantly limits interpretation. The authors should either provide the WT data or clearly explain why these groups were excluded.

**Response 1:** We thank the reviewer’s thoughtful question on this point. Thank you for pointing this out. In preliminary Western blot analyses, human p-tau signal was undetectable in WT mice, as expected given the absence of the P301S tau transgene (Additional figure. 3). Therefore, we did not include WT samples in the Western blot analysis. Also, we restricted our analysis to PS19 mice to assess the pharmacological impact of omipalisib within a pathologically relevant setting.

**Additional Figure. 3** Expression of phosphorylated tau in WT and PS19 mice. (A) Western blot band of phosphorylated tau and GAPDH. (B) The quantification of Ser262 was normalized to GAPDH. (C) The quantification of Ser404 was normalized to GAPDH. (D) The quantification of Ser396 was normalized to GAPDH. (E) The quantification of AT8 was normalized to GAPDH. Statistical analysis was performed using the student’s *t* test. Data represent the mean ± SEM. ***p* < 0.01, ****p* < 0.001, and *****p* < 0.0001 compared to the WT group.

**Question 2:** Please provide a rationale for selecting the dosage of Omipalisib used in the in vivo experiments?

**Response 2:** We appreciate the reviewer for raising this question. To select the appropriate omipalisib dosage, we referred to the published article [Med Sci Monit. 2020 Aug 17:26:e927106]. Following that result, we performed a pilot study testing 1 and 3 mg/kg of omipalisib and monitored survival and body weight. In our protocol, 3 of 7 mice treated with 3 mg/kg of omipalisib died, whereas there was no dead mouse in the 1 mg/kg group and body weight in this group also didn’t significantly change (Additional figure. 2). Based on these observations, we selected 1 mg/kg of omipalisib for our main experiment.

**Additional Figure. 2** Effects of omipalisib on survival and body weight in mice. (A) Survival of mice treated with omipalisib at 1 mg/kg and 3 mg/kg for 7 days. (B) Body weight changes in mice treated with 1 mg/kg omipalisib for 7 days. Data represent the mean ± SEM.

**Question 3:** The in vitro experiments employed SH-SY5Y cells expressing P301L (0N4R) mutant tau (SH-Tau; P30705), whereas the in vivo experiments used PS19 mice expressing the P301S (1N4R) mutant tau. Could the difference between 0N4R and 1N4R isoforms lead to divergent outcomes, and how do the authors interpret this potential discrepancy?

**Response 3:** Thank you for your question. We agree that the in vitro and in vivo systems use different tau isoforms/mutations (SH-SY5Y cells expressing 0N4R-P30L and PS19 mice expressing 1N4R-P301S). Although the N-terminal insert (0N vs 1N) can influence aggregation propensity and kinetics [J Biol Chem. 2012 Apr 26;287(24):20711–20719], both constructs are in the 4R background and harbor P301X substitutions that have been shown to impair microtubule binding and increase aggregation [J Biol Chem. 2019 Oct 24;294(48):18488–18503; Biochim Biophys Acta. 2005 Jan 3;1739(2-3):331-54]. Thus, we expect the core biology underlying our readouts to be conserved across models and we have focused on the directional concordance of key phenotypes (e.g. reduction in p-tau and enhancement of autophagy) rather than absolute magnitudes. We have carefully added a discussion of isoform-dependent effects in the revised manuscripts as follows:

- Page 14, line 5 in the discussion

The second limitation of our study is that the in vitro and in vivo systems express different N-terminal tau isoforms (0N4R vs 1N4R). Although N-terminal differences are known to affect tau aggregation kinetics (29), both models express 4R tau harboring P301X mutations, which are known to impair microtubule binding and promote aggregation (30, 31). Thus, we focused on directional consistency of the main phenotypes (e.g. reduction in p-tau and enhancement of autophagy) across models. Future studies using matched isoform/mutation combinations would strengthen translational interpretation.

**Question 4:** Is Omipalisib capable of crossing the blood-brain barrier (BBB)? Experimental or pharmacokinetic evidence supporting is CNS permeability should be provided or discussed.

**Response 4:** We appreciate the reviewer’s comment. Pharmacokinetic study has shown that omipalisib (GSK2126458) is measurable in the brain after oral administration at 10 mg/kg in wild-type mice, suggesting partial BBB permeability under normal conditions [J Pharmacol Exp Ther. 2016 Feb;356(2):251-9]. Additionally, omipalisib has a logP value of approximately 3.2, one hydrogen bond donor, and seven hydrogen bond acceptors, which fall within the physicochemical range generally associated with BBB permeability. Therefore, we think that omipalisib crossed the BBB in our experimental protocol as well, although the actual brain exposure after intraperitoneal injection at 1 mg/kg requires further confirmation.
