# Supplementary material for: Omipalisib reduces hyperphosphorylated tau protein by modulating mTOR-autophagy pathway
Source: PLoS One. 2026 Jun 23;21(6):e0352120. doi: 10.1371/journal.pone.0352120 (PMC13289877; doi:10.1371/journal.pone.0352120)
Supplement: Attachment — Submitted filename: PLOS_ONE_--25-23710-R1.docx [file pone.0352120.s009.docx]

**Review Comments to the Author**

The authors have substantially revised the manuscript and have addressed the majority of the concerns raised in the previous round of review. The study presents technically sound in vitro and in vivo data demonstrating that omipalisib treatment is associated with reduced tau phosphorylation and improved spatial memory performance in a tauopathy mouse model. Overall, the work is suitable for publication in *PLOS ONE* after **minor revisions**, primarily related to mechanistic phrasing, statistical rigor, and editorial consistency.

Below I outline specific points that should be addressed to further strengthen the manuscript and ensure full compliance with journal standards.

**1. Autophagy mechanism: evidence remains supportive, not definitive**

**What was added:**
The authors included a 3-methyladenine (3-MA) co-treatment experiment (Fig. S3) showing that omipalisib-mediated reduction of Ser262 p-tau is blocked when autophagy is pharmacologically inhibited.

- 3-MA inhibits early autophagosome formation but also targets **class III PI3K**, which is mechanistically upstream of mTOR and overlaps with the pharmacological target of omipalisib.
- Autophagic flux was **not directly assessed** using lysosomal inhibitors (e.g., bafilomycin A1, chloroquine) or genetic approaches (ATG5/ATG7 knockdown).
- LC3B-II changes are reported but in several cases do **not reach statistical significance** (e.g., Fig. 2D reports p-values > 0.05).

**Specific example of overstatement:**

- Results section (Section 3.2) and Discussion state that omipalisib “activates autophagy” or “decreases p-tau through autophagy activation.”

**Required revision:**

- Replace strong causal language with qualified phrasing such as:
  - “consistent with involvement of autophagy”
  - “suggests that autophagy contributes to p-tau reduction”
- Ensure that this tempered language is used consistently in the **Results, Discussion, and Conclusion**, not only in the limitations paragraph.

**2. In vivo mechanistic chain is only partially demonstrated**

**What was addressed:**
The authors added in vivo evidence showing reduced **p-mTOR/mTOR** levels in PS19 mouse brain following omipalisib treatment (Fig. 4).

**What remains missing:**

- No direct assessment of downstream **autophagy markers in brain tissue** (e.g., LC3-II accumulation, p62 degradation).
- As a result, the mechanistic sequence
  *mTOR inhibition → autophagy activation → tau clearance*
  is not fully demonstrated in vivo.

**Specific example:**

- Section 3.3 states that p-tau reduction is mediated by mTOR signaling, but autophagy activation in the brain is inferred rather than measured.

**Required revision:**

- Explicitly acknowledge in the Discussion that **in vivo autophagy activation is inferred** from upstream signaling and in vitro data.
- Avoid implying that autophagy activation in brain tissue has been directly demonstrated.

**3. Behavioral statistics: repeated-measures issue must be clarified**

**Concern:**

- Morris water maze acquisition involves **repeated measurements of the same animals across days**.
- The manuscript states that two-way ANOVA was used, but it is unclear whether a **repeated-measures ANOVA or mixed-effects model** was applied.

**Specific example:**

- Figure 5A (escape latency across training days) appears to represent longitudinal data but the statistical model is not clearly specified.

**Required revision:**

- Explicitly state in the Methods and/or figure legend whether:
  - two-way **repeated-measures** ANOVA, or
  - a **mixed-effects model**
    was used.
- If standard two-way ANOVA without repeated-measures structure was applied, this should be corrected or justified.

**4. Overstatement of non-significant results**

**Specific example:**

- In Section 3.3, soluble Ser262 p-tau is described as “reduced,” despite a reported p-value of **p = 0.1175**, which is not statistically significant.

**Why this matters:**

- Describing non-significant results as decreases overstates the data and may mislead readers.

**Required revision:**

- Rephrase such statements to:
  - “showed a non-significant trend toward reduction.”
  - or report them descriptively without implying statistical significance.

**5. Data Availability statement inconsistency**

**Issue:**

- One section states that “all relevant data are within the manuscript and its Supporting Information files.”
- A later Data Availability section states that datasets are “available from the corresponding author on reasonable request.”
- *PLOS ONE* generally does **not accept ‘available upon request’** unless a justified exception applies.

**Required revision:**

- Harmonize the Data Availability Statement to clearly indicate that all data are publicly available in the manuscript and supplementary materials, or provide a policy-compliant justification if any restriction applies.
- LC3B interpretation is still internally inconsistent (Figure-level issue)

What the figures show (your Fig. 2D):

- LC3B-II/LC3B-I increases numerically
- BUT error bars are large
- AND p-values are non-significant across timepoints

The problem:

- The manuscript visually *suggests* autophagy activation via LC3B
- The statistics do not support that claim
- This creates a visual–statistical contradiction

Required minor fix:

- In the figure legend and Results text, explicitly state:

“LC3B-II/LC3B-I ratios showed a non-significant increase, whereas p62 levels were significantly reduced.”

-p62 decrease alone ≠ autophagic flux (conceptual precision)

What you show:

- Strong, clean, statistically significant p62 reduction (Fig. 2C)
- This is the best autophagy-related result in the paper

The problem:

- p62 decrease can occur via transcriptional or proteasomal mechanisms
- Without flux controls, p62 ≠ proof of increased autophagic degradation

Required minor fix:

- Add one sentence in Results or Discussion:

“Although p62 reduction is consistent with enhanced autophagic degradation, p62 levels alone do not establish autophagic flux.”

- 3-MA experiment: mechanistic ambiguity must be acknowledged

What the figure shows (3-MA panel):

- 3-MA blocks omipalisib-induced Ser262 reduction
- This is supportive, not definitive

The problem:

- 3-MA is a PI3K inhibitor
- Omipalisib is a PI3K/mTOR inhibitor
- You are blocking a pathway *upstream of the drug target*

Required minor fix:

- Add one explicit limitation sentence, e.g.:

“Because 3-MA also inhibits class III PI3K, which overlaps with omipalisib’s upstream signaling, this experiment supports but does not definitively establish autophagy-specific causality.”

That single sentence neutralizes a major conceptual objection.

- In vivo autophagy is still inferred, not shown (Figure logic gap)

What you show:

- p-mTOR ↓ in PS19 brain (good)
- p-tau ↓ in soluble and insoluble fractions (good)

What is missing:

- No LC3B or p62 in brain tissue

The problem:

- You implicitly claim:
  p-mTOR ↓ → autophagy ↑ → tau ↓
- But you only show first and last steps

Required minor fix:

- Add a clarifying sentence in Results or Discussion:

“Downstream autophagy markers were not directly measured in brain tissue, and thus in vivo autophagy activation is inferred rather than directly demonstrated.”

- Soluble tau Ser262 is overstated (again, but now figure-driven)

What the figure shows:

- Soluble Ser262:
  - Downward trend
  - Large SEM
  - Not statistically significant

The problem:

- Text says “reduced”
- Figure says “trend”

Required minor fix:

- Replace *“reduced”* with:
  - “did not reach statistical significance”
  - or “showed a non-significant decrease”

- Morris Water Maze: statistics vs design mismatch

What the figure shows:

- Longitudinal acquisition curves (Days 1–7)
- Same animals measured repeatedly

The problem:

- If this was analyzed as standard two-way ANOVA, that is wrong
- Must be two-way repeated-measures ANOVA or mixed-effects

Required minor fix:

- Explicitly state in Methods:

“Escape latency across training days was analyzed using two-way repeated-measures ANOVA.”

Even if they already did it—it must be written.

- WT controls absent from tau WB figures (interpretation clarity)

What the figures show:

- Tau WB figures only compare PS19 vs PS19+Omi
- WT lanes are absent (though explained elsewhere)

The problem:

- Readers skimming figures will wonder:

“Is tau absent in WT? Why not shown?”

Required minor fix:

- Add one sentence in figure legend or Results:

“WT mice were not included in tau immunoblot analyses because human p-tau was undetectable.”

- Behavioral rescue magnitude vs tau reduction not discussed

What the data show:

- Partial behavioral rescue
- Significant but not complete normalization
- Tau reduction is also partial

The problem:

- No discussion of dose–effect proportionality
- No acknowledgement that cognitive rescue is incomplete

Required minor fix:

- Add one sentence in Discussion:

“Although omipalisib significantly improved spatial memory, performance did not fully normalize to WT levels, consistent with partial reduction of pathological tau.”

-BBB claim is still speculative

What is stated:

- Omipalisib is “likely BBB-permeable” based on logP and prior studies

The problem:

- No direct brain concentration measurements at 1 mg/kg i.p.
- BBB penetration is assumed, not demonstrated

Required minor fix:

- Add one qualifying sentence:

“Actual brain exposure at the dose and route used here was not directly measured.”

- Title still slightly over-claims mechanism

Current title:

“reduces hyperphosphorylated tau protein via activating autophagy through mTOR inhibition”

The problem:

- “via activating autophagy” implies proven causality

Safer minor revision:

- “…associated with mTOR inhibition and autophagy-related signaling”
- OR keep title but soften in Abstract/Conclusion (editor may insist)
- The Discussion would benefit from citing relevant prior studies by Yadikar et al. demonstrating pharmacological modulation of tau hyperphosphorylation and emphasizing that tau reduction reflects broader proteostasis dynamics rather than a single clearance pathway (e.g., Yadikar et al., *PLOS ONE* 2020; *Molecular Neurobiology* 2023; *Int. J. Mol. Sci.* 2024). Inclusion of this work would strengthen contextual interpretation of tau reduction, mechanistic caution, and the relationship between molecular and behavioral outcomes.
