# Supplementary material for: Omipalisib reduces hyperphosphorylated tau protein by modulating mTOR-autophagy pathway
Source: PLoS One. 2026 Jun 23;21(6):e0352120. doi: 10.1371/journal.pone.0352120 (PMC13289877; doi:10.1371/journal.pone.0352120)
Supplement: Attachment — Submitted filename: Response_to_Reviewers_auresp_2.docx [file pone.0352120.s010.docx]

**Dear Editor and Reviewers,**

We appreciate your kind and wise comments, the opportunity to revise our work, and your review of this work. We have responded to the comments as follows and revised the manuscript after carefully considering your suggestions. In the revised manuscript, the changed parts were written in blue font.

**----------------------------------------------------------------------------------------------------------------**

**Regarding the reviewer #1’s comments**

The authors present a technically solid in vitro and in vivo study examining the effects of the dual PI3K/mTOR inhibitor omipalisib on tau phosphorylation and cognitive performance in a tauopathy model. The experimental execution is generally sound, and the data support an association between omipalisib treatment, reduced phosphorylated tau, and improved spatial memory. However, several substantive issues remain that preclude acceptance at this stage and require major revision, primarily related to statistical rigor, data availability compliance, and mechanistic interpretation. Addressing these points will substantially improve the rigor, transparency, and interpretative accuracy of the manuscript.

**Question 1:** First, the statistical analysis of the Morris water maze acquisition data requires correction or explicit clarification. Acquisition curves represent repeated measurements of the same animals across training days, yet the manuscript does not clearly state whether a repeated-measures ANOVA or mixed-effects model was used. Application of standard two-way ANOVA without accounting for within-subject dependence would be inappropriate and must be addressed explicitly.

**Response 1:** We thank the reviewer for this important comment. The Morris water maze acquisition data were analyzed using a mixed-effects model in GraphPad Prism. Treatment group and training day were included as fixed effects, and mouse was treated as a random effect to account for repeated measurements across days. The interaction group and day were included in the analysis. We have carefully added this clarification to the revised manuscripts and revised figure caption as follows:

- Page 10, line 1 in the methods

For behavioral data obtained from the Morris water maze acquisition trials, mixed-effects model was used. Treatment group and training day were included as fixed effects, and mouse was specified as a random effect. The group × day interaction was incorporated into the model. Multiple comparisons were performed within each training day to compare treatment groups.

- Page 6, line 6 in the figure 5 caption

Morris water maze acquisition data were analyzed using a mixed-effects model.

**Question 2:** Second, multiple results are overstated relative to their statistical support. For example, soluble Ser262 phosphorylated tau is described as “reduced” despite not reaching statistical significance (p > 0.05). Such statements should be revised to accurately reflect non-significant trends rather than definitive effects.

**Response 2:** We appreciate the reviewer’s comment. We have carefully revised the description of soluble Ser262 phosphorylated tau to more accurately reflect in the revised manuscripts as follows:

- Page 11, line 19 in the results

Omipalisib treatment significantly reduced the levels of soluble p-tau at Ser396 (*p* = 0.0041) and AT8 (*p* = 0.0001), whereas soluble p-tau at Ser262 did not show a significant reduction (*p* = 0.1175) (Fig. 3A–D).

**Question 3:** Third, although the authors added supportive experiments using 3-methyladenine, the mechanistic conclusion that omipalisib reduces tau phosphorylation via autophagy activation remains insufficiently demonstrated. Autophagic flux was not directly measured, LC3B-II changes are largely non-significant, and 3-MA has PI3K-related off-target effects that overlap with the drug’s mechanism. Accordingly, causal language throughout the manuscript (including the title, Results, and Conclusion) should be tempered to reflect association rather than definitive pathway mediation.

**Response 3:** We thank the reviewer for pointing out a potential lack of clarity in our study. We acknowledge that autophagic flux was not directly measured, LC3B-II changes were largely non-significant, and that 3-MA may have PI3K-related off-target effects. Accordingly, we have revised the manuscript to temper causal language throughout the title, results, and discussion, and now describe the effects of omipalisib as associative rather than definitive. We reflected these revisions in the revised manuscript as follows:

- Page 1, line 1 in the manuscript

Omipalisib reduces hyperphosphorylated tau protein by modulating mTOR-autophagy pathway

- Page 10, line 10 in the results

Omipalisib reduces phosphorylated tau in SH-Tau cells

- Page 10, line 21 in the results

Omipalisib modulates autophagy-related protein via the mTOR pathway in SH-Tau cells

- Page 11, line 11 in the results

These data indicated that omipalisib decreased the levels of accumulated p-tau by modulating ALP signaling through mTOR inhibition.

- Page 14, line 20 in the discussion

These results suggest that the reduction of p-tau by omipalisib may be associated with changes in mTOR-mediated autophagy signaling.

- Page 15, line 5 in the discussion

Therefore, our findings indicate a potential association between omipalisib treatment and changes in autophagy markers, but additional experiments are required to fully demonstrate autophagic flux.

- Page 15, line 17 in the discussion

These therapeutic effects of omipalisib appear to involve modulation of the mTOR-mediated autophagy signals.

- Page 15, line 18 in the discussion

Although autophagic flux was not directly measured, our findings suggest a potential association between omipalisib treatment and pathways involved in tau clearance.

**Question 4:** Fourth, in vivo autophagy activation is inferred but not directly shown, as downstream autophagy markers (e.g., LC3-II or p62) were not assessed in brain tissue. This limitation should be stated explicitly in the Results and Discussion to avoid overinterpretation.

**Response 4:** We appreciate the reviewer’s comments. We agree that in vivo autophagy activation was inferred rather than directly demonstrated. Accordingly, we have explicitly stated this limitation in the discussion, noting that downstream autophagy markers such as LC3-II and p62 were not directly assessed in brain tissue as follows:

- Page 15, line 3 in the discussion

In addition, in vivo autophagy activation was inferred from upstream signaling changes, as downstream autophagy markers such as LC3-II and p62 were not directly assessed in brain tissue.

**Question 5:** Fifth, the Data Availability statement must be corrected to comply with PLOS ONE policy. Conflicting language regarding data inclusion versus availability “upon reasonable request” should be resolved, and all underlying data should be clearly and publicly accessible or appropriately justified.

**Response 5:** Thank you for the reviewer’s comment. We have revised the Data availability statement to comply with the PLOS ONE policy and clarified that all relevant data underlying the findings are available within the manuscript and its supporting information files.

- Page 17, line 13 in the manuscript

Data availability statement

All relevant data are within the manuscript and its supporting information files.

**Question 6:** Finally, the Discussion would benefit from citing relevant prior studies that contextualize tau phosphorylation reduction within broader proteostasis and functional outcome frameworks (e.g., Yadikar et al., PLOS ONE 2020; Molecular Neurobiology 2023; International Journal of Molecular Sciences 2024), particularly given the partial behavioral rescue observed and the absence of direct autophagic flux measurements.

**Response6:** Thank you for the reviewer’s helpful suggestion. We have incorporated the suggested references (Yadikar et al., PLOS ONE 2020; Molecular Neurobiology 2023; International Journal of Molecular Sciences 2024) and revised the Discussion to place our findings within the broader context of tau kinase regulation and proteostasis networks. We have carefully added discussion regarding the partial behavioral rescue observed in our study as follows:

- Page 13, line 22 in the discussion

This reduction in p-tau aligns with established frameworks in which targeting tau-related kinase pathways mitigate pathological tau accumulation (29). Furthermore, such regulation is important for maintaining cellular proteostasis, as abnormal tau modifications can disrupt broader protein homeostasis networks (30).

- Page 12, line 19 in the results

It should also be noted that the partial behavioral rescue observed in our study may reflect the complex molecular signatures and diverse pathological pathways involved in tauopathy, suggesting that multiple mechanisms likely contribute to functional outcomes in tauopathy models (16).

**Regarding the reviewer #2’s comments**

Overall, the authors have addressed most of my previous concerns and I am satisfied with the revised manuscript. However, I would like to ask that the responses to my Comments 1, 2, and 4 be more clearly and explicitly reflected in the revised manuscript itself, not only in the rebuttal letter. Below is my specific comments.

**Question 1:** I have one remaining concern regarding the new data provided in response to Comment 1. In the western blot, the Tau5 signal appears to be absent in the WT mouse samples. Since Tau5 is expected to detect total tau and should be detectable in WT brain lysates under standard conditions, this pattern is unexpected and makes the blot difficult to interpret. The authors should clarify and explain why Tau5 is not detectable in the WT lanes. If possible, providing an additional representative blot or further validation would help to resolve this issue and strengthen confidence in the western blot data.

**Response 1:** We thank the reviewer for this point. Tau 5 is a pan-tau antibody that recognizes both mouse and human tau, and therefore endogenous mouse tau is expected to be detectable in WT lysates. In our data, Tau5 signal is not absent in WT lysates but appears weaker than that observed in PS19 mice. This difference likely reflects the overexpression of the human P301S tau transgene in the PS19 model relative to endogenous mouse tau [Neuron. 2007 Feb 1;53(3):337-51.]. In our blot, a faint band corresponding to endogenous mouse tau is detectable in WT brain lysates (~50-55 kDa). In contrast, PS19 mice exhibit a much stronger band around ~64 kDa, reflecting the high expression of the human P301S tau protein (Additional figure. 1). Similar banding patterns have also been reported using tau transgenic mouse models [Neurobiol Dis. 2025 Nov;216:107145; Int J Mol Sci. 2022 Jan 10;23(2):718.].

**Additional Figure. 1** Expression of total tau (tau5) in brain of WT and PS19 mice.

**Question 2:** For the comments 1 and 2, it would be helpful if the additional figures could be presented as Supplmentary figure, so that readers can refer to these results.

**Response 2:** We thank the reviewer for this helpful suggestion. The additional data and representative images have been included as supplementary figures 2 and 7 in the revised manuscripts as follows:

- Page 6, line 17 in the methods

We conducted a pilot study evaluating omipalisib at 1 and 3 mg/kg and monitored survival and body weight. In the 3 mg/kg group, 3 of 7 mice died, whereas no mortality was observed in the 1 mg/kg group, and body weight did not show a significant change (Fig. S2). Based on these observations, 1 mg/kg was selected for the main experiment.

- Page 14, line 18 in the discussion

Although omipalisib has the potential to influence the PI3K-Akt-glycogen synthase kinase 3β (GSK3β) pathway, p-GSK3β (Ser9) levels were not significantly altered (Fig. S7).

**Question 3:** It would be helpful if authors include the Response related to Comment 4 in the manuscript such as in the discussion section where appropriate.

**Response 3:** We appreciate the reviewer’s comment. We have carefully added a discussion addressing the reported brain penetration and physicochemical properties of omipalisib and clarified the limitation regarding the absence of direct brain exposure measurements in our study in the revised manuscripts as follows:

- Page 14, line 9 in the discussion

Pharmacokinetic study has shown that omipalisib is detectable in the brain after oral administration at 10 mg/kg in normal mice, suggesting partial BBB permeability under normal conditions (32). Additionally, omipalisib has a logP value of approximately 3.2, one hydrogen bond donor, and seven hydrogen bond acceptors, properties that fall within the physicochemical range generally associated with BBB permeability. These findings indicate that omipalisib has the potential to penetrate the BBB.
