# Supplementary material for: Omipalisib reduces hyperphosphorylated tau protein by modulating mTOR-autophagy pathway
Source: PLoS One. 2026 Jun 23;21(6):e0352120. doi: 10.1371/journal.pone.0352120 (PMC13289877; doi:10.1371/journal.pone.0352120)
Supplement: Attachment — Submitted filename: Response_to_Reviewers_auresp_3.docx [file pone.0352120.s011.docx]

**Dear Editor and Reviewers,**

We appreciate your kind and wise comments, the opportunity to revise our work, and your review of this work. We have responded to the comments as follows and revised the manuscript after carefully considering your suggestions. In the revised manuscript, the changed parts were written in blue font.

**----------------------------------------------------------------------------------------------------------------**

**Regarding the reviewer #1’s comments**

The authors have substantially improved the manuscript and addressed most of the previous concerns. The study now presents technically sound in vitro and in vivo data supporting an association between omipalisib treatment, reduced tau phosphorylation, and improved spatial memory performance in the PS19 tauopathy model. The clarification of the Morris water maze statistics and the inclusion of a policy-compliant data availability statement are appreciated. Overall, the manuscript is suitable for publication in PLOS ONE after minor revision.

**Question 1:** Mechanistic phrasing remains too strong in the Abstract. Although the title and parts of the Discussion are now appropriately tempered, the Abstract still states that omipalisib decreases p-tau “through autophagy activation by mTOR inhibition.” This wording remains stronger than the evidence supports. Because autophagic flux was not directly measured, please revise the Abstract to use qualified language such as “associated with modulation of mTOR/autophagy-related signaling” or “consistent with a contribution of autophagy.”

**Response 1:** We thank the reviewer for this important comment. We have revised the abstract to use more cautious language, describing the effects of omipalisib as being associated with mTOR/autophagy-related signaling rather than definitively mediated by autophagy activation. We revised the abstract in the revised manuscript as follows:

- Page 3, line 17 in the abstract

Overall, these results show that omipalisib decreases the expression of p-tau by modulation mTOR-autophagy pathway, resulting in the amelioration of spatial memory deficits.

**Question 2:** LC3B interpretation should be stated more carefully. The p62 result is clear and statistically supportive, whereas the LC3B-II/LC3B-I increase appears non-significant. Please state this explicitly in the Results and/or Figure 2 legend and avoid implying definitive autophagy activation from LC3B alone.

**Response 2:** We appreciate the reviewer’s comment. We have revised the results to clearly distinguish between the statistically significant decrease in p62 levels and the non-significant changes in LC3B-II/LC3B-I ratios, and to avoid overinterpretation of LC3B data. We reflected these revisions in the revised manuscript as follows:

- Page 11, line 6 in the results

We observed a significant decrease in p62 levels following omipalisib treatment (3 hr, p = 0.0021; 6 hr, p = 0.0004; 12 hr, p < 0.0001; 24 hr, p < 0.0001, Fig. 2C). Although LC3B-II/LC3B-I ratios showed a trend towards an increase, these changes were not significant (3 hr, p = 0.1237; 6 hr, p = 0.2776; 12 hr, p = 0.0857; 24 hr, p = 0.7543, Fig. 2D).

**Question 3:** The 3-MA experiment should be framed as supportive, not definitive.
Please add one sentence acknowledging that 3-MA also inhibits class III PI3K and therefore does not establish autophagy-specific causality independent of upstream PI3K signaling.

**Response 3:** We thank the reviewer for pointing out a potential lack of clarity in our study. In response, we have revised the Discussion to clarify that the 3-MA experiment provides supportive evidence rather than definitive proof, and to note that 3-MA also inhibits class III PI3K, which may confound interpretation of autophagy-specific effects. We revised the discussion in the revised manuscript as follows:

- Page 15, line 2 in the discussion

Although 3-MA was used to assess whether omipalisib-induced effects are modulated by autophagy, autophagic flux was not directly confirmed using lysosomal inhibitors or genetic approaches, and additional studies are required to further clarify this mechanism.

**Question 4:** Please keep the in vivo mechanism qualified throughout. The Discussion appropriately notes that downstream autophagy markers were not measured in brain tissue. Please ensure that no remaining text implies direct demonstration of in vivo autophagy activation.

**Response 4:** We appreciate the reviewer’s comments. We have revised the relevant sections of the Discussion to avoid overstating the in vivo mechanism and to maintain appropriately cautious interpretation. We clarified that the involvement of autophagy in vivo was inferred from upstream signaling changes, as downstream markers such as LC3-II and p62 were not directly evaluated in brain tissue. We have incorporated the corresponding revisions in the revised manuscript as follows:

- Page 15, line 5 in the discussion

In addition, possible involvement of autophagy in vivo was inferred from upstream signaling changes, as downstream autophagy markers such as LC3-II and p62 were not directly assessed in brain tissue.

**Question 5:** The BBB discussion would also benefit from one explicit sentence stating that brain exposure at the dose and route used here was not directly measured.

**Response 5:** Thank you for the reviewer’s comment. We have added a clarifying statement in the Discussion to indicate that brain exposure at the dose and route used in this study was not directly measured. The revised text has been incorporated in the revised manuscript as follows

- Page 14, line 13 in the discussion

These findings indicate that omipalisib has the potential to penetrate the BBB, but additional confirmation would be valuable with the condition used in this study.

**Question 6:** Please move the WT/Tau5 clarification into the manuscript or supplementary material. The rebuttal explanation is helpful, but readers should not need the rebuttal letter to understand the immunoblot context. A short clarifying sentence in the Results, figure legend, or a supplementary figure would resolve this.

**Response 6:** Thank you for the reviewer’s helpful suggestion. We have added a clarifying sentence to the figure legend to ensure that the immunoblot context is clearly understood without reference to the rebuttal. We revised the figure caption in the revised figure as follows:

- Page 4, line 4 in the figure 3 caption

P-tau levels were measured in PS19 mice only, as the analysis focused on human tau expressed in the PS19 mice.

**Question 7:** Please audit references and editorial consistency carefully. Reference 35 appears unrelated to tau biology and may be erroneous.

**Response 7:** We thank the reviewer for this insightful comment. We have carefully reviewed the reference list and corrected the inappropriate citation, including Reference 35, to ensure that all references are accurate and relevant to tau biology. In addition, we have checked the consistency of the references throughout the manuscript

**Question 8:** Please also ensure that funding/financial disclosure information is fully consistent across the manuscript, acknowledgments, and submission metadata.

**Response 8:** We thank the reviewer for this comment. In accordance with the journal's submission guidelines, funding information was removed from the manuscript and is instead provided in the submission metadata. We confirm that the funding/financial disclosure information has been entered consistently and accurately in the system (see attached screenshot of the relevant webpage). If preferred, we would be happy to include this information in the manuscript as well.

**Regarding the reviewer #2’s comments**

The authors have satisfactorily addressed all my previous comments. I have no further concerns and endorse this manuscript for publication.
