# Supplementary material for: Omipalisib reduces hyperphosphorylated tau protein by modulating mTOR-autophagy pathway
Source: PLoS One. 2026 Jun 23;21(6):e0352120. doi: 10.1371/journal.pone.0352120 (PMC13289877; doi:10.1371/journal.pone.0352120)
Supplement: Attachment — Submitted filename: Response_to_Reviewers_auresp_4.docx [file pone.0352120.s012.docx]

**Dear Editor,**

We thank the Editorial Office for this request. We have completed the humane endpoints checklist and uploaded it as file type “Other” with the revised submission.

We have revised the methods to include additional details on animal monitoring, and predefined humane endpoint criteria. We clarified that animals were monitored at least once daily and that humane endpoints included severe lethargy, inability to access food or water, marked weight loss (>20% of baseline body weight), persistent hunched posture, or severe respiratory distress. Animals meeting these criteria were immediately removed from the study and euthanized according to the approved animal protocol.

We also clarified that death was no used as a planned experimental endpoint. In the pilot tolerability study, 3 of 7 mice in the 3 mg/kg group reached humane endpoint criteria and were euthanized, whereas no animals in the 1 mg/kg group reached humane endpoints or died. During the subsequent 2-month treatment study using 1 mg/kg omipalisib, no animals reached humane endpoint criteria, required euthanasia, or died.
