# Supplementary material for: Omipalisib reduces hyperphosphorylated tau protein by modulating mTOR-autophagy pathway
Source: PLoS One. 2026 Jun 23;21(6):e0352120. doi: 10.1371/journal.pone.0352120 (PMC13289877; doi:10.1371/journal.pone.0352120)
Supplement: Peer review history

# Peer review: Decision Letter 0

**DOI:** 10.1371/journal.pone.0352120.r001

**Authors:** Firas Kobeissy

9 Sep 2025

PONE-D-25-23710

Omipalisib reduces hyperphosphorylated tau protein via activating mTOR-mediated autophagy

PLOS ONE

Dear Dr. Lee,

Thank you for submitting your manuscript to PLOS ONE. After careful consideration, we feel that it has merit but does not fully meet PLOS ONE’s publication criteria as it currently stands. Therefore, we invite you to submit a revised version of the manuscript that addresses the points raised during the review process.

Please submit your revised manuscript by Oct 24 2025 11:59PM. If you will need more time than this to complete your revisions, please reply to this message or contact the journal office at plosone@plos.org. When you're ready to submit your revision, log on to https://www.editorialmanager.com/pone/ and select the 'Submissions Needing Revision' folder to locate your manuscript file.

Please include the following items when submitting your revised manuscript:

- A rebuttal letter that responds to each point raised by the academic editor and reviewer(s). You should upload this letter as a separate file labeled 'Response to Reviewers'.
- A marked-up copy of your manuscript that highlights changes made to the original version. You should upload this as a separate file labeled 'Revised Manuscript with Track Changes'.
- An unmarked version of your revised paper without tracked changes. You should upload this as a separate file labeled 'Manuscript'.

If you would like to make changes to your financial disclosure, please include your updated statement in your cover letter. Guidelines for resubmitting your figure files are available below the reviewer comments at the end of this letter.

If applicable, we recommend that you deposit your laboratory protocols in protocols.io to enhance the reproducibility of your results. Protocols.io assigns your protocol its own identifier (DOI) so that it can be cited independently in the future. For instructions see: https://journals.plos.org/plosone/s/submission-guidelines#loc-laboratory-protocols. Additionally, PLOS ONE offers an option for publishing peer-reviewed Lab Protocol articles, which describe protocols hosted on protocols.io. Read more information on sharing protocols at https://plos.org/protocols?utm_medium=editorial-email&utm_source=authorletters&utm_campaign=protocols.

We look forward to receiving your revised manuscript.

Kind regards,

Firas H Kobeissy, PhD

Academic Editor

PLOS ONE

Journal requirements:

When submitting your revision, we need you to address these additional requirements.

1. Please ensure that your manuscript meets PLOS ONE's style requirements, including those for file naming. The PLOS ONE style templates can be found at

https://journals.plos.org/plosone/s/file?id=wjVg/PLOSOne_formatting_sample_main_body.pdf and

https://journals.plos.org/plosone/s/file?id=ba62/PLOSOne_formatting_sample_title_authors_affiliations.pdf

2. To comply with PLOS ONE submissions requirements, in your Methods section, please provide additional information regarding the experiments involving animals and ensure you have included details on (1) methods of sacrifice, and (2) efforts to alleviate suffering.

3. In the online submission form, you indicated that [The datasets used and analyzed during the current study are available from the corresponding author upon reasonable request. Data cannot be shared publicly due to internal institutional regulations.].

All PLOS journals now require all data underlying the findings described in their manuscript to be freely available to other researchers, either 1. In a public repository, 2. Within the manuscript itself, or 3. Uploaded as supplementary information.

This policy applies to all data except where public deposition would breach compliance with the protocol approved by your research ethics board. If your data cannot be made publicly available for ethical or legal reasons (e.g., public availability would compromise patient privacy), please explain your reasons on resubmission and your exemption request will be escalated for approval.

4. When completing the data availability statement of the submission form, you indicated that you will make your data available on acceptance. We strongly recommend all authors decide on a data sharing plan before acceptance, as the process can be lengthy and hold up publication timelines. Please note that, though access restrictions are acceptable now, your entire data will need to be made freely accessible if your manuscript is accepted for publication. This policy applies to all data except where public deposition would breach compliance with the protocol approved by your research ethics board. If you are unable to adhere to our open data policy, please kindly revise your statement to explain your reasoning and we will seek the editor's input on an exemption. Please be assured that, once you have provided your new statement, the assessment of your exemption will not hold up the peer review process.

5. PLOS requires an ORCID iD for the corresponding author in Editorial Manager on papers submitted after December 6th, 2016. Please ensure that you have an ORCID iD and that it is validated in Editorial Manager. To do this, go to ‘Update my Information’ (in the upper left-hand corner of the main menu), and click on the Fetch/Validate link next to the ORCID field. This will take you to the ORCID site and allow you to create a new iD or authenticate a pre-existing iD in Editorial Manager.

6. PLOS ONE now requires that authors provide the original uncropped and unadjusted images underlying all blot or gel results reported in a submission’s figures or Supporting Information files. This policy and the journal’s other requirements for blot/gel reporting and figure preparation are described in detail at https://journals.plos.org/plosone/s/figures#loc-blot-and-gel-reporting-requirements and https://journals.plos.org/plosone/s/figures#loc-preparing-figures-from-image-files. When you submit your revised manuscript, please ensure that your figures adhere fully to these guidelines and provide the original underlying images for all blot or gel data reported in your submission. See the following link for instructions on providing the original image data: https://journals.plos.org/plosone/s/figures#loc-original-images-for-blots-and-gels.

In your cover letter, please note whether your blot/gel image data are in Supporting Information or posted at a public data repository, provide the repository URL if relevant, and provide specific details as to which raw blot/gel images, if any, are not available. Email us at plosone@plos.org if you have any questions.

7. Please include a separate caption for each figure in your manuscript.

8. If the reviewer comments include a recommendation to cite specific previously published works, please review and evaluate these publications to determine whether they are relevant and should be cited. There is no requirement to cite these works unless the editor has indicated otherwise.

Additional Editor Comments:

Reviewer #1:

Reviewer #2:

[Note: HTML markup is below. Please do not edit.]

Reviewers' comments:

Reviewer's Responses to Questions

**Comments to the Author**

1. Is the manuscript technically sound, and do the data support the conclusions?

The manuscript must describe a technically sound piece of scientific research with data that supports the conclusions. Experiments must have been conducted rigorously, with appropriate controls, replication, and sample sizes. The conclusions must be drawn appropriately based on the data presented.

Reviewer #1: No

Reviewer #2: Yes

**********

2. Has the statistical analysis been performed appropriately and rigorously?

Reviewer #1: No

Reviewer #2: Yes

**********

3. Have the authors made all data underlying the findings in their manuscript fully available?

The PLOS Data policy requires authors to make all data underlying the findings described in their manuscript fully available without restriction, with rare exception (please refer to the Data Availability Statement in the manuscript PDF file). The data should be provided as part of the manuscript or its supporting information, or deposited to a public repository. For example, in addition to summary statistics, the data points behind means, medians and variance measures should be available. If there are restrictions on publicly sharing data—e.g. participant privacy or use of data from a third party—those must be specified.

Reviewer #1: No

Reviewer #2: Yes

**********

4. Is the manuscript presented in an intelligible fashion and written in standard English?

PLOS ONE does not copyedit accepted manuscripts, so the language in submitted articles must be clear, correct, and unambiguous. Any typographical or grammatical errors should be corrected at revision, so please note any specific errors here.

Reviewer #1: Yes

Reviewer #2: Yes

**********

5. Review Comments to the Author

Please use the space provided to explain your answers to the questions above. You may also include additional comments for the author, including concerns about dual publication, research ethics, or publication ethics. (Please upload your review as an attachment if it exceeds 20,000 characters)

Reviewer #1: Summary

This manuscript explores the effects of Omipalisib, a dual PI3K/mTOR inhibitor, on tau pathology in cellular and PS19 mouse models. It shows that Omipalisib reduces phosphorylated tau (p-tau) by activating autophagy, correlating with improved memory performance in mice. The study is well-designed and addresses a relevant target in neurodegenerative disease. However, key mechanistic claims lack direct validation, and several aspects of clarity, terminology, and data interpretation must be revised.

Major Comments

Autophagy Not Directly Proven

LC3 and p62 levels are shown, but autophagic flux is not assessed.

Recommend adding bafilomycin/lysosomal inhibitor experiments or using Atg5/Atg7 knockdown to confirm flux.

If not feasible, tone down conclusions and acknowledge this limitation.

Mechanistic Specificity

Omipalisib is inaccurately described as a "selective mTOR inhibitor" — it's a dual PI3K/mTOR inhibitor.

Please revise all text accordingly and discuss implications (e.g., PI3K→Akt→GSK3β feedback on tau kinases).

In Vivo Mechanism Confirmation Needed

No evidence of mTOR inhibition or autophagy activation in mouse brain.

Recommend measuring p-mTOR, LC3-II, or p62 in PS19 mouse brain lysates.

Histological Validation Missing

Western blot shows tau reduction; immunohistochemistry for p-tau (e.g., AT8), and neuroinflammation (GFAP, Iba1) would strengthen the case.

Clarify Behavioral Analysis

Use repeated-measures ANOVA for MWM training.

Confirm motor performance was unaffected (distance, speed) and report blinding.

Safety Observations in Mice

Report any weight changes, health issues, or behavioral effects of chronic Omipalisib treatment.

Improve Discussion

Compare to other autophagy inducers (rapamycin, trehalose, metformin, BEZ235).

Acknowledge Omipalisib’s systemic toxicity risks and translational challenges.

Minor Comments

Title: Rephrase to “...via mTOR inhibition” (mTOR inhibits autophagy, not activates it).

Clarify “ALS” (autophagy–lysosome system) to avoid confusion with amyotrophic lateral sclerosis.

Western blots: Show representative images in figures or supplementary files.

Reference: Add more on dual inhibitors in tauopathy (e.g., BEZ235).

Methods: Clarify drug formulation, injection route, randomization/blinding, sex of mice.

Statistical details: Provide exact p-values where possible; confirm correction for multiple comparisons.

Language edits: Fix typos (e.g., “preformed” → “performed”), and check for grammar and clarity.

Overall Recommendation

This is a strong study with therapeutic implications, but requires a major revision to justify its conclusions. Addressing the autophagy mechanism, clarifying drug specificity, and expanding the in vivo validation will substantially improve the manuscript. With these revisions, the manuscript will be suitable for publication in PLOS ONE.

Reviewer #2: This manuscript investigates the effects of Omipalisib, a dual PI3K/mTOR inhibitor, on tau pathology using both cellular (SH-SY5Y tau mutant) and animal (PS19) models. The authors demonstrate that Omipalisib reduces hyperphosphorylated tau, activates autophagy, and alleviates cognitive impairment. The topic is relevant and of translational interest. However, there are several critical issues that must be addressed before this work is suitable for publication.

Major Comments

1. In Figure 3 (tau Western blots) and Supplementary Fig. S2 (Neuroinflammation data), WT groups are missing, despite being described in the Methods section (four groups: WT+vehicle, WT+Omipalisib, PS19+vehicle, PS19+Omipalisib). The absence of WT controls significantly limits interpretation. The authors should either provide the WT data or clearly explain why these groups were excluded.

2. Please provide a rationale for selecting the dosage of Omipalisib used in the in vivo experiments?

3. The in vitro experiments employed SH-SY5Y cells expressing P301L (0N4R) mutant tau (SH-Tau; P30705), whereas the in vivo experiments used PS19 mice expressing the P301S (1N4R) mutant tau. Could the difference between 0N4R and 1N4R isoforms lead to divergent outcomes, and how do the authors interpret this potential discrepancy?

4. Is Omipalisib capable of crossing the blood-brain barrier (BBB)? Experimental or pharmacokinetic evidence supporting is CNS permeability should be provided or discussed.

**********

6. PLOS authors have the option to publish the peer review history of their article (what does this mean?). If published, this will include your full peer review and any attached files.

If you choose “no”, your identity will remain anonymous but your review may still be made public.

**Do you want your identity to be public for this peer review?**   For information about this choice, including consent withdrawal, please see our Privacy Policy.

Reviewer #1: **Yes:**   Hamad Yadikar

Reviewer #2: No

**********

[NOTE: If reviewer comments were submitted as an attachment file, they will be attached to this email and accessible via the submission site. Please log into your account, locate the manuscript record, and check for the action link "View Attachments". If this link does not appear, there are no attachment files.]

While revising your submission, please upload your figure files to the Preflight Analysis and Conversion Engine (PACE) digital diagnostic tool, https://pacev2.apexcovantage.com/. PACE helps ensure that figures meet PLOS requirements. To use PACE, you must first register as a user. Registration is free. Then, login and navigate to the UPLOAD tab, where you will find detailed instructions on how to use the tool. If you encounter any issues or have any questions when using PACE, please email PLOS at figures@plos.org. Please note that Supporting Information files do not need this step.

# Author comment: Author response to Decision Letter 1

**DOI:** 10.1371/journal.pone.0352120.r002

9 Dec 2025

We sincerely appreciate the valuable comments and constructive feedback provided by you and the reviewers. These thoughtful suggestions have significantly improved our revised manuscript. We look forward to continuing the process toward publication in PLOS One.

The revised manuscript has been carefully reviewed, and all necessary changes have been made in accordance with the reviewers’ suggestions. Detailed responses to all comments are provided and attached herewith.

Attachment Submitted filename: Response to Reviewers.docx

# Peer review: Decision Letter 1

**DOI:** 10.1371/journal.pone.0352120.r003

**Authors:** Firas Kobeissy

18 Feb 2026

PONE-D-25-23710R1

Omipalisib reduces hyperphosphorylated tau protein via activating autophagy through mTOR inhibition

PLOS One

Dear Dr. Lee,

Thank you for submitting your manuscript to PLOS ONE. After careful consideration, we feel that it has merit but does not fully meet PLOS ONE’s publication criteria as it currently stands. Therefore, we invite you to submit a revised version of the manuscript that addresses the points raised during the review process.

Please submit your revised manuscript by Apr 03 2026 11:59PM. If you will need more time than this to complete your revisions, please reply to this message or contact the journal office at plosone@plos.org. When you're ready to submit your revision, log on to https://www.editorialmanager.com/pone/ and select the 'Submissions Needing Revision' folder to locate your manuscript file.

Please include the following items when submitting your revised manuscript:

- A letter that responds to each point raised by the academic editor and reviewer(s). You should upload this letter as a separate file labeled 'Response to Reviewers'.
- A marked-up copy of your manuscript that highlights changes made to the original version. You should upload this as a separate file labeled 'Revised Manuscript with Track Changes'.
- An unmarked version of your revised paper without tracked changes. You should upload this as a separate file labeled 'Manuscript'.

If you would like to make changes to your financial disclosure, please include your updated statement in your cover letter. Guidelines for resubmitting your figure files are available below the reviewer comments at the end of this letter.

If applicable, we recommend that you deposit your laboratory protocols in protocols.io to enhance the reproducibility of your results. Protocols.io assigns your protocol its own identifier (DOI) so that it can be cited independently in the future. For instructions see: https://journals.plos.org/plosone/s/submission-guidelines#loc-laboratory-protocols. Additionally, PLOS ONE offers an option for publishing peer-reviewed Lab Protocol articles, which describe protocols hosted on protocols.io. Read more information on sharing protocols at https://plos.org/protocols?utm_medium=editorial-email&utm_source=authorletters&utm_campaign=protocols.

We look forward to receiving your revised manuscript.

Kind regards,

Firas H Kobeissy, PhD

Academic Editor

PLOS One

Journal Requirements:

If the reviewer comments include a recommendation to cite specific previously published works, please review and evaluate these publications to determine whether they are relevant and should be cited. There is no requirement to cite these works unless the editor has indicated otherwise.

[Note: HTML markup is below. Please do not edit.]

Reviewers' comments:

Reviewer's Responses to Questions

**Comments to the Author**

1. If the authors have adequately addressed your comments raised in a previous round of review and you feel that this manuscript is now acceptable for publication, you may indicate that here to bypass the “Comments to the Author” section, enter your conflict of interest statement in the “Confidential to Editor” section, and submit your "Accept" recommendation.

Reviewer #1: (No Response)

Reviewer #2: All comments have been addressed

**********

2. Is the manuscript technically sound, and do the data support the conclusions?

The manuscript must describe a technically sound piece of scientific research with data that supports the conclusions. Experiments must have been conducted rigorously, with appropriate controls, replication, and sample sizes. The conclusions must be drawn appropriately based on the data presented.

Reviewer #1: Yes

Reviewer #2: Yes

**********

3. Has the statistical analysis been performed appropriately and rigorously?

Reviewer #1: No

Reviewer #2: Yes

**********

4. Have the authors made all data underlying the findings in their manuscript fully available?

The PLOS Data policy requires authors to make all data underlying the findings described in their manuscript fully available without restriction, with rare exception (please refer to the Data Availability Statement in the manuscript PDF file). The data should be provided as part of the manuscript or its supporting information, or deposited to a public repository. For example, in addition to summary statistics, the data points behind means, medians and variance measures should be available. If there are restrictions on publicly sharing data—e.g. participant privacy or use of data from a third party—those must be specified.

Reviewer #1: No

Reviewer #2: Yes

**********

5. Is the manuscript presented in an intelligible fashion and written in standard English?

PLOS ONE does not copyedit accepted manuscripts, so the language in submitted articles must be clear, correct, and unambiguous. Any typographical or grammatical errors should be corrected at revision, so please note any specific errors here.

Reviewer #1: Yes

Reviewer #2: Yes

**********

6. Review Comments to the Author

Please use the space provided to explain your answers to the questions above. You may also include additional comments for the author, including concerns about dual publication, research ethics, or publication ethics. (Please upload your review as an attachment if it exceeds 20,000 characters)

Reviewer #1: The authors present a technically solid in vitro and in vivo study examining the effects of the dual PI3K/mTOR inhibitor omipalisib on tau phosphorylation and cognitive performance in a tauopathy model. The experimental execution is generally sound, and the data support an association between omipalisib treatment, reduced phosphorylated tau, and improved spatial memory. However, several substantive issues remain that preclude acceptance at this stage and require major revision, primarily related to statistical rigor, data availability compliance, and mechanistic interpretation.

First, the statistical analysis of the Morris water maze acquisition data requires correction or explicit clarification. Acquisition curves represent repeated measurements of the same animals across training days, yet the manuscript does not clearly state whether a repeated-measures ANOVA or mixed-effects model was used. Application of standard two-way ANOVA without accounting for within-subject dependence would be inappropriate and must be addressed explicitly.

Second, multiple results are overstated relative to their statistical support. For example, soluble Ser262 phosphorylated tau is described as “reduced” despite not reaching statistical significance (p > 0.05). Such statements should be revised to accurately reflect non-significant trends rather than definitive effects.

Third, although the authors added supportive experiments using 3-methyladenine, the mechanistic conclusion that omipalisib reduces tau phosphorylation via autophagy activation remains insufficiently demonstrated. Autophagic flux was not directly measured, LC3B-II changes are largely non-significant, and 3-MA has PI3K-related off-target effects that overlap with the drug’s mechanism. Accordingly, causal language throughout the manuscript (including the title, Results, and Conclusion) should be tempered to reflect association rather than definitive pathway mediation.

Fourth, in vivo autophagy activation is inferred but not directly shown, as downstream autophagy markers (e.g., LC3-II or p62) were not assessed in brain tissue. This limitation should be stated explicitly in the Results and Discussion to avoid overinterpretation.

Fifth, the Data Availability statement must be corrected to comply with PLOS ONE policy. Conflicting language regarding data inclusion versus availability “upon reasonable request” should be resolved, and all underlying data should be clearly and publicly accessible or appropriately justified.

Finally, the Discussion would benefit from citing relevant prior studies that contextualize tau phosphorylation reduction within broader proteostasis and functional outcome frameworks (e.g., Yadikar et al., PLOS ONE 2020; Molecular Neurobiology 2023; International Journal of Molecular Sciences 2024), particularly given the partial behavioral rescue observed and the absence of direct autophagic flux measurements.

Addressing these points will substantially improve the rigor, transparency, and interpretative accuracy of the manuscript.

Reviewer #2: Overall, the authors have addressed most of my previous concerns and I am satisfied with the revised manuscript. However, I would like to ask that the responses to my Comments 1, 2, and 4 be more clearly and explicitly reflected in the revised manuscript itself, not only in the rebuttal letter. Below is my specific comments.

1. I have one remaining concern regarding the new data provided in response to Comment 1. In the western blot, the Tau5 signal appears to be absent in the WT mouse samples. Since Tau5 is expected to detect total tau and should be detectable in WT brain lysates under standard conditions, this pattern is unexpected and makes the blot difficult to interpret. The authors should clarify and explain why Tau5 is not detectable in the WT lanes. If possible, providing an additional representative blot or further validation would help to resolve this issue and strengthen confidence in the western blot data.

2. For the comments 1 and 2, it would be helpful if the additional figures could be presented as Supplmentary figure, so that readers can refer to these results.

3. It would be helpful if authors include the Response related to Comment 4 in the manuscript such as in the discussion section where appropriate.

**********

7. PLOS authors have the option to publish the peer review history of their article (what does this mean?). If published, this will include your full peer review and any attached files.

If you choose “no”, your identity will remain anonymous but your review may still be made public.

**Do you want your identity to be public for this peer review?**   For information about this choice, including consent withdrawal, please see our Privacy Policy.

Reviewer #1: **Yes:**   Hamad Yadikar

Reviewer #2: No

**********

[NOTE: If reviewer comments were submitted as an attachment file, they will be attached to this email and accessible via the submission site. Please log into your account, locate the manuscript record, and check for the action link "View Attachments". If this link does not appear, there are no attachment files.]

To ensure your figures meet our technical requirements, please review our figure guidelines: https://journals.plos.org/plosone/s/figures

You may also use PLOS’s free figure tool, NAAS, to help you prepare publication quality figures: https://journals.plos.org/plosone/s/figures#loc-tools-for-figure-preparation.

NAAS will assess whether your figures meet our technical requirements by comparing each figure against our figure specifications.

Attachment Submitted filename: PLOS_ONE_--25-23710-R1.docx

# Author comment: Author response to Decision Letter 2

**DOI:** 10.1371/journal.pone.0352120.r004

10 Mar 2026

The revised manuscript has been rechecked, and the necessary changes have been made in accordance with the reviewer’s suggestions. The responses to all comments have been prepared and attached herewith.

Attachment Submitted filename: Response_to_Reviewers_auresp_2.docx

# Peer review: Decision Letter 2

**DOI:** 10.1371/journal.pone.0352120.r005

**Authors:** Firas Kobeissy

5 Apr 2026

PONE-D-25-23710R2

Omipalisib reduces hyperphosphorylated tau protein by modulating mTOR-autophagy pathway

PLOS One

Dear Dr. Lee,

Thank you for submitting your manuscript to PLOS ONE. After careful consideration, we feel that it has merit but does not fully meet PLOS ONE’s publication criteria as it currently stands. Therefore, we invite you to submit a revised version of the manuscript that addresses the points raised during the review process.

Please submit your revised manuscript by May 20 2026 11:59PM. If you will need more time than this to complete your revisions, please reply to this message or contact the journal office at plosone@plos.org. When you're ready to submit your revision, log on to https://www.editorialmanager.com/pone/ and select the 'Submissions Needing Revision' folder to locate your manuscript file.

Please include the following items when submitting your revised manuscript:

- A letter that responds to each point raised by the academic editor and reviewer(s). You should upload this letter as a separate file labeled 'Response to Reviewers'.
- A marked-up copy of your manuscript that highlights changes made to the original version. You should upload this as a separate file labeled 'Revised Manuscript with Track Changes'.
- An unmarked version of your revised paper without tracked changes. You should upload this as a separate file labeled 'Manuscript'.

If you would like to make changes to your financial disclosure, please include your updated statement in your cover letter. Guidelines for resubmitting your figure files are available below the reviewer comments at the end of this letter.

If applicable, we recommend that you deposit your laboratory protocols in protocols.io to enhance the reproducibility of your results. Protocols.io assigns your protocol its own identifier (DOI) so that it can be cited independently in the future. For instructions see: https://journals.plos.org/plosone/s/submission-guidelines#loc-laboratory-protocols. Additionally, PLOS ONE offers an option for publishing peer-reviewed Lab Protocol articles, which describe protocols hosted on protocols.io. Read more information on sharing protocols at https://plos.org/protocols?utm_medium=editorial-email&utm_source=authorletters&utm_campaign=protocols.

As the corresponding author, your ORCID iD is verified in the submission system and will appear in the published article. PLOS supports the use of ORCID, and we encourage all coauthors to register for an ORCID iD and use it as well. Please encourage your coauthors to verify their ORCID iD within the submission system before final acceptance, as unverified ORCID iDs will not appear in the published article. *Only*   the individual author can complete the verification step; PLOS staff *cannot*   verify ORCID iDs on behalf of authors.

We look forward to receiving your revised manuscript.

Kind regards,

Firas H Kobeissy, PhD

Academic Editor

PLOS One

Journal Requirements:

1. If the reviewer comments include a recommendation to cite specific previously published works, please review and evaluate these publications to determine whether they are relevant and should be cited. There is no requirement to cite these works unless the editor has indicated otherwise.

2. Please review your reference list to ensure that it is complete and correct. If you have cited papers that have been retracted, please include the rationale for doing so in the manuscript text, or remove these references and replace them with relevant current references. Any changes to the reference list should be mentioned in the rebuttal letter that accompanies your revised manuscript. If you need to cite a retracted article, indicate the article’s retracted status in the References list and also include a citation and full reference for the retraction notice.

[Note: HTML markup is below. Please do not edit.]

Reviewers' comments:

Reviewer's Responses to Questions

**Comments to the Author**

1. If the authors have adequately addressed your comments raised in a previous round of review and you feel that this manuscript is now acceptable for publication, you may indicate that here to bypass the “Comments to the Author” section, enter your conflict of interest statement in the “Confidential to Editor” section, and submit your "Accept" recommendation.

Reviewer #1: All comments have been addressed

Reviewer #2: All comments have been addressed

**********

2. Is the manuscript technically sound, and do the data support the conclusions?

The manuscript must describe a technically sound piece of scientific research with data that supports the conclusions. Experiments must have been conducted rigorously, with appropriate controls, replication, and sample sizes. The conclusions must be drawn appropriately based on the data presented.

Reviewer #1: Yes

Reviewer #2: Yes

**********

3. Has the statistical analysis been performed appropriately and rigorously?

Reviewer #1: (No Response)

Reviewer #2: Yes

**********

4. Have the authors made all data underlying the findings in their manuscript fully available?

The PLOS Data policy requires authors to make all data underlying the findings described in their manuscript fully available without restriction, with rare exception (please refer to the Data Availability Statement in the manuscript PDF file). The data should be provided as part of the manuscript or its supporting information, or deposited to a public repository. For example, in addition to summary statistics, the data points behind means, medians and variance measures should be available. If there are restrictions on publicly sharing data—e.g. participant privacy or use of data from a third party—those must be specified.

Reviewer #1: Yes

Reviewer #2: Yes

**********

5. Is the manuscript presented in an intelligible fashion and written in standard English?

PLOS ONE does not copyedit accepted manuscripts, so the language in submitted articles must be clear, correct, and unambiguous. Any typographical or grammatical errors should be corrected at revision, so please note any specific errors here.

Reviewer #1: Yes

Reviewer #2: Yes

**********

6. Review Comments to the Author

Please use the space provided to explain your answers to the questions above. You may also include additional comments for the author, including concerns about dual publication, research ethics, or publication ethics. (Please upload your review as an attachment if it exceeds 20,000 characters)

Reviewer #1: Comments to the Authors

The authors have substantially improved the manuscript and addressed most of the previous concerns. The study now presents technically sound in vitro and in vivo data supporting an association between omipalisib treatment, reduced tau phosphorylation, and improved spatial memory performance in the PS19 tauopathy model. The clarification of the Morris water maze statistics and the inclusion of a policy-compliant data availability statement are appreciated. Overall, the manuscript is suitable for publication in PLOS ONE after minor revision.

Mechanistic phrasing remains too strong in the Abstract.

Although the title and parts of the Discussion are now appropriately tempered, the Abstract still states that omipalisib decreases p-tau “through autophagy activation by mTOR inhibition.” This wording remains stronger than the evidence supports. Because autophagic flux was not directly measured, please revise the Abstract to use qualified language such as “associated with modulation of mTOR/autophagy-related signaling” or “consistent with a contribution of autophagy.”

LC3B interpretation should be stated more carefully.

The p62 result is clear and statistically supportive, whereas the LC3B-II/LC3B-I increase appears non-significant. Please state this explicitly in the Results and/or Figure 2 legend and avoid implying definitive autophagy activation from LC3B alone.

The 3-MA experiment should be framed as supportive, not definitive.

Please add one sentence acknowledging that 3-MA also inhibits class III PI3K and therefore does not establish autophagy-specific causality independent of upstream PI3K signaling.

Please keep the in vivo mechanism qualified throughout.

The Discussion appropriately notes that downstream autophagy markers were not measured in brain tissue. Please ensure that no remaining text implies direct demonstration of in vivo autophagy activation. The BBB discussion would also benefit from one explicit sentence stating that brain exposure at the dose and route used here was not directly measured.

Please move the WT/Tau5 clarification into the manuscript or supplementary material.

The rebuttal explanation is helpful, but readers should not need the rebuttal letter to understand the immunoblot context. A short clarifying sentence in the Results, figure legend, or a supplementary figure would resolve this.

Please audit references and editorial consistency carefully.

Reference 35 appears unrelated to tau biology and may be erroneous. Please also ensure that funding/financial disclosure information is fully consistent across the manuscript, acknowledgments, and submission metadata.

Reviewer #2: The authors have satisfactorily addressed all my previous comments. I have no further concerns and endorse this manuscript for publication.

**********

7. PLOS authors have the option to publish the peer review history of their article (what does this mean?). If published, this will include your full peer review and any attached files.

If you choose “no”, your identity will remain anonymous but your review may still be made public.

**Do you want your identity to be public for this peer review?**   For information about this choice, including consent withdrawal, please see our Privacy Policy.

Reviewer #1: **Yes:**   Hamad Yadikar

Reviewer #2: No

**********

[NOTE: If reviewer comments were submitted as an attachment file, they will be attached to this email and accessible via the submission site. Please log into your account, locate the manuscript record, and check for the action link "View Attachments". If this link does not appear, there are no attachment files.]

To ensure your figures meet our technical requirements, please review our figure guidelines: https://journals.plos.org/plosone/s/figures

You may also use PLOS’s free figure tool, NAAS, to help you prepare publication quality figures: https://journals.plos.org/plosone/s/figures#loc-tools-for-figure-preparation.

NAAS will assess whether your figures meet our technical requirements by comparing each figure against our figure specifications.

# Author comment: Author response to Decision Letter 3

**DOI:** 10.1371/journal.pone.0352120.r006

15 Apr 2026

We thank you and the reviewers for your thoughtful suggestions and insights. The revised manuscript has benefited from these insightful suggestions. We look forward to working with you and the reviewers to move this manuscript closer to publication in PLOS One. The revised manuscript has been rechecked, and the necessary changes have been made in accordance with the reviewer’s suggestions. The responses to all comments have been prepared and attached herewith.

Attachment Submitted filename: Response_to_Reviewers_auresp_3.docx

# Editor report: Decision Letter 3

**DOI:** 10.1371/journal.pone.0352120.r007

**Authors:** Firas Kobeissy

13 May 2026

PONE-D-25-23710R3

Omipalisib reduces hyperphosphorylated tau protein by modulating mTOR-autophagy pathway

PLOS One

Dear Dr. Lee,

Thank you for submitting your manuscript to PLOS ONE. After careful consideration, we feel that it has merit but does not fully meet PLOS ONE’s publication criteria as it currently stands. Therefore, we invite you to submit a revised version of the manuscript that addresses the points raised during the review process.

=============

Request from the Editorial Office:

We note that your study design may include death of a regulated animal as a likely outcome or planned experimental endpoint. At this time, we request that you please report additional details in your Methods section regarding animal care and use for the survival study, as per our editorial guidelines (http://journals.plos.org/plosone/s/submission-guidelines#loc-humane-endpoints).

For easy reference, we have attached a checklist that may be relevant for your submission. Please complete all items on the checklist at the following link: http://journals.plos.org/plosone/s/file?id=bb1d/plos-one-humane-endpoints-checklist.docx

Please upload the completed checklist as file type “Other” when resubmitting your manuscript. This document is for internal journal use only and will not be published if your article is accepted. We very much appreciate your attention to these requests and support of improved reporting standards in PLOS One submissions.

=============

Please submit your revised manuscript by Jun 26 2026 11:59PM. If you will need more time than this to complete your revisions, please reply to this message or contact the journal office at plosone@plos.org. When you're ready to submit your revision, log on to https://www.editorialmanager.com/pone/ and select the 'Submissions Needing Revision' folder to locate your manuscript file.

Please include the following items when submitting your revised manuscript:

- A letter that responds to each point raised by the academic editor and reviewer(s). You should upload this letter as a separate file labeled 'Response to Reviewers'.
- A marked-up copy of your manuscript that highlights changes made to the original version. You should upload this as a separate file labeled 'Revised Manuscript with Track Changes'.
- An unmarked version of your revised paper without tracked changes. You should upload this as a separate file labeled 'Manuscript'.

If you would like to make changes to your financial disclosure, please include your updated statement in your cover letter. Guidelines for resubmitting your figure files are available below the reviewer comments at the end of this letter.

If applicable, we recommend that you deposit your laboratory protocols in protocols.io to enhance the reproducibility of your results. Protocols.io assigns your protocol its own identifier (DOI) so that it can be cited independently in the future. For instructions see: https://journals.plos.org/plosone/s/submission-guidelines#loc-laboratory-protocols. Additionally, PLOS ONE offers an option for publishing peer-reviewed Lab Protocol articles, which describe protocols hosted on protocols.io. Read more information on sharing protocols at https://plos.org/protocols?utm_medium=editorial-email&utm_source=authorletters&utm_campaign=protocols.

As the corresponding author, your ORCID iD is verified in the submission system and will appear in the published article. PLOS supports the use of ORCID, and we encourage all coauthors to register for an ORCID iD and use it as well. Please encourage your coauthors to verify their ORCID iD within the submission system before final acceptance, as unverified ORCID iDs will not appear in the published article. *Only*   the individual author can complete the verification step; PLOS staff *cannot*   verify ORCID iDs on behalf of authors.

We look forward to receiving your revised manuscript.

Kind regards,

Firas H Kobeissy, PhD

Academic Editor

PLOS One

Journal Requirements:

If the reviewer comments include a recommendation to cite specific previously published works, please review and evaluate these publications to determine whether they are relevant and should be cited. There is no requirement to cite these works unless the editor has indicated otherwise.

Please review your reference list to ensure that it is complete and correct. If you have cited papers that have been retracted, please include the rationale for doing so in the manuscript text, or remove these references and replace them with relevant current references. Any changes to the reference list should be mentioned in the rebuttal letter that accompanies your revised manuscript. If you need to cite a retracted article, indicate the article’s retracted status in the References list and also include a citation and full reference for the retraction notice.

[Note: HTML markup is below. Please do not edit.]

[NOTE: If reviewer comments were submitted as an attachment file, they will be attached to this email and accessible via the submission site. Please log into your account, locate the manuscript record, and check for the action link "View Attachments". If this link does not appear, there are no attachment files.]

To ensure your figures meet our technical requirements, please review our figure guidelines: https://journals.plos.org/plosone/s/figures

You may also use PLOS’s free figure tool, NAAS, to help you prepare publication quality figures: https://journals.plos.org/plosone/s/figures#loc-tools-for-figure-preparation.

NAAS will assess whether your figures meet our technical requirements by comparing each figure against our figure specifications.

# Author comment: Author response to Decision Letter 4

**DOI:** 10.1371/journal.pone.0352120.r008

23 May 2026

We thank you and the reviewers for your thoughtful suggestions and insights. We have carefully revised the manuscript in response to all comments. The revised manuscript has benefited from these insightful suggestions.

Attachment Submitted filename: Response_to_Reviewers_auresp_4.docx

# Editor report: Decision Letter 4

**DOI:** 10.1371/journal.pone.0352120.r009

**Authors:** Firas Kobeissy

4 Jun 2026

Omipalisib reduces hyperphosphorylated tau protein by modulating mTOR-autophagy pathway

PONE-D-25-23710R4

Dear Dr. Lee,

We’re pleased to inform you that your manuscript has been judged scientifically suitable for publication and will be formally accepted for publication once it meets all outstanding technical requirements.

Within one week, you’ll receive an e-mail detailing the required amendments. When these have been addressed, you’ll receive a formal acceptance letter and your manuscript will be scheduled for publication.

An invoice will be generated when your article is formally accepted. Please note, if your institution has a publishing partnership with PLOS and your article meets the relevant criteria, all or part of your publication costs will be covered. Please make sure your user information is up-to-date by logging into Editorial Manager at Editorial Manager® and clicking the ‘Update My Information' link at the top of the page. For questions related to billing, please contact billing support.

If your institution or institutions have a press office, please notify them about your upcoming paper to help maximize its impact. If they’ll be preparing press materials, please inform our press team as soon as possible -- no later than 48 hours after receiving the formal acceptance. Your manuscript will remain under strict press embargo until 2 pm Eastern Time on the date of publication. For more information, please contact onepress@plos.org.

Kind regards,

Firas H Kobeissy, PhD

Academic Editor

PLOS One

Additional Editor Comments (optional):

Reviewers' comments:

# Editor report: Acceptance letter

**DOI:** 10.1371/journal.pone.0352120.r010

**Authors:** Firas Kobeissy

PONE-D-25-23710R4

PLOS One

Dear Dr. Lee,

I'm pleased to inform you that your manuscript has been deemed suitable for publication in PLOS One. Congratulations! Your manuscript is now being handed over to our production team.

At this stage, our production department will prepare your paper for publication. This includes ensuring the following:

* All references, tables, and figures are properly cited

* All relevant supporting information is included in the manuscript submission,

* There are no issues that prevent the paper from being properly typeset

You will receive further instructions from the production team, including instructions on how to review your proof when it is ready. Please keep in mind that we are working through a large volume of accepted articles, so please give us a few days to review your paper and let you know the next and final steps.

Lastly, if your institution or institutions have a press office, please let them know about your upcoming paper now to help maximize its impact. If they'll be preparing press materials, please inform our press team within the next 48 hours. Your manuscript will remain under strict press embargo until 2 pm Eastern Time on the date of publication. For more information, please contact onepress@plos.org.

You will receive an invoice from PLOS for your publication fee after your manuscript has reached the completed accept phase. If you receive an email requesting payment before acceptance or for any other service, this may be a phishing scheme. Learn how to identify phishing emails and protect your accounts at https://explore.plos.org/phishing.

If we can help with anything else, please email us at customercare@plos.org.

Thank you for submitting your work to PLOS ONE and supporting open access.

Kind regards,

PLOS ONE Editorial Office Staff

on behalf of

Dr. Firas H Kobeissy

Academic Editor

PLOS One
